# Synergistic impact of osseointegration and multifunctionality on functionally graded ceramic composites for bone healing applications

**DOI:** 10.1038/s41598-025-94835-z

**Published:** 2025-04-11

**Authors:** Rasha A. Youness, Essam B. Mostafa, Mohammed A. Taha

**Affiliations:** 1 Spectroscopy Department, National Research Centre, El Buhouth St, Dokki, Giza, 12622 Egypt; 2 Faculty of Engineering, Benha National University (BNU), Al Obour City, Egypt; 3 Solid State Physics Department, National Research Centre, El Buhouth St, Dokki, Giza, 12622 Egypt; 4 Pharos University in Alexandria, Canal Mahmoudiah St, Smouha, Alexandria Egypt

**Keywords:** Functionally graded composites, Osseointegration, Biocompatibility, Multifunctional performance, Bone healing applications, Biophysics, Materials science

## Abstract

In this study, a functionally graded composite (FGC) sample was fabricated by layering five layers of hydroxyapatite (HA), silicon carbide (SiC), and copper oxide (CuO) nanoparticles. The osseointegration ability of all prepared FGC layers was assessed using simulated body fluid (SBF), and an investigation was performed using scanning electron microscopy (SEM). The physical, mechanical, electrical, and dielectric properties were evaluated before and after immersion in the SBF solution. Additionally, the antibacterial efficacy and biocompatibility of these layers were examined. Sintered layers exhibit porosity values ​​ranging from 5 to 10%, similar to compact bone, which is essential for effective osseointegration. SEM images showed good bioactive behavior across all FGC layers. The measured ultimate strength of the samples was 77.30, 81.54, 85.80, 93.29, and 102.82 MPa due to the successive increase in SiC and CuO content of the samples. This makes it mainly identical to cortical bone, sparing the bone from a stress-shielded effect. Soaking the produced layers with SBF did not impact their mechanical properties, indicating that their biological activity and mechanical properties are compatible. However, their electrical properties altered somewhat after soaking in SBF. Notably, the sample with the highest SiC and CuO content exhibited a 75% reduction in weight loss after applying a load of 10 N. Also, CuO inclusion in the studied layers led to a significant inhibition in *S. aureus* and *E. coli* bacterial growth up to 23 and 16 mm, respectively, without a noticeable cytotoxic effect. Based on these findings, the developed FGC sample and its layers are appropriate for bone healing applications.

## Introduction

Orthopedic surgery continues to face significant difficulties in the skeletal restoration of bone abnormalities for load-bearing applications, prompting scientists to meet these challenges to solve this problem^1^. Functionally graded composites (FGCs) are particularly interesting in orthopedics because of their distinctive characteristics, including stiffness, strength, flexibility, and toughness^2,3^. FGCs have a gradient or heterogeneous material distribution. The strength of the areas under more significant amounts of stress can be increased to produce FGCs with superior mechanical properties and minimal weight^4^. As graded materials’ chemical composition or microstructure progressively shifts in one or more desired directions, there is a smooth and gradual change in properties from one side to the other^5^. Because human bone is a typical biological FGC, FGCs are designed to mimic the inherent variability in bone tissues; they are the best choice for bone replacement. For instance, bone density and mechanical characteristics decline from the cancellous interior to the compact outside area^6^.

For this reason, the ideal orthopedic FGC requirement can be met by improving the design of the structure and materials. FGCs have been made using a variety of processes, including physical vapor deposition (PVD), chemical vapor deposition (CVD), centrifugal casting, tape casting, and powder metallurgy (PM)^7^. The powder technique is primarily employed in FGC fabrication to get the finest internal structure. However, fabrication methods such as conventional sintering, stir casting, infiltration, plasma spraying, and spark plasma have also been studied^8^.

One potential use for biomaterials in biomedicine is to incorporate electrically conductive materials that can improve tissue healing. This application can be utilized in a variety of situations. Nevertheless, there is a lack of enthusiasm for pursuing electrically conductive materials for orthopedic applications. Fukada and Yasuda’s discovery in the 1950s that bone exhibits electrical conductivity ignited the initial interest in this area. By applying mechanical pressure on the bone from different angles, researchers discovered that this action generated electrical signals within the bone. These signals created an internal electric field that facilitated the growth of osteoblasts and sped up the process of osseointegration^9^. Additionally, a few investigations revealed that after implantation, the repair of bone occupancy at the wounded bone and the osteoconduction process were significantly impacted by the accumulation of electrical charges on bone replacement material^10^. Given this data, it is strongly advised that materials with good electrical characteristics be obtained for orthopedic applications.

In FGC preparations, hydroxyapatite (HA; Ca_10_(PO_4_)_6_(OH)_2_) is a desirable biomaterial because of its exceptional qualities, such as its high degree of biocompatibility and its striking likeness to natural human bone. In the crystal structure of HA, the partial substitution of carbonate (CO_3_^2−^) groups for phosphate (PO_4_^3−^) groups results in B-type carbonated hydroxyapatite (B-CHA), which offers remarkable biological performance and improved solubility when combined with nanoscale HA preparation. However, despite these incredible properties, its weak mechanical qualities restrict its medicinal applications. As a result, creating composites or morphological changes is the only way to improve HA’s mechanical properties^11,12^.

The remarkable oxidation, high strength, excellent chemical resistance, thermal stability, hardness, melting point, and other exceptional properties of silicon carbide (SiC), a non − oxide ceramic, give SiC its outstanding advantages. This makes it useful for abrasion, cutting, and high-temperature electrical devices^13,14^. Because of these appealing properties, SiC may effectively cure bone problems. Unfortunately, this material’s widespread use is limited by its lack of bioactivity and inability to promote bone formation, osseointegration, and growth. It must be combined with another biologically active phase for bone replacement applications to overcome these constraints and provide the requisite biological, chemical, and physical characteristics^15^.

Strong electrical and optical qualities, low cost, and thermal stability are only some of copper oxide’s (CuO) desirable physicochemical characteristics. Unquestionably, CuO is essential for many vital bodily functions, including maintaining bone volume, accelerating wound healing, and supporting enzymatic processes. It also supports the operation of the immune system and the synthesis of nucleic acids. Meanwhile, it also has antibacterial, anticancer, and antioxidant properties^16^. Given the outstanding qualities of each material mentioned above, a good combination should provide the biological and physicochemical characteristics of biomaterials used in orthopedic applications^17^.

Although numerous studies^18–22^ have examined the mechanical properties of FGCs for use in orthopedic applications, none have examined the effects of a bioactive layer that forms after soaking an FGC sample and its layers in SBF for 14 days. Our work fills that gap by investigating the impact of this layer on the physical, mechanical, and electrical attributes of FGCs. We also evaluate its antimicrobial properties, wear resistance, and bone cell compatibility, offering crucial new information on the potential of FGCs as long-lasting, biocompatible implants.

## Materials and methods

### Materials

In this study, analytical graded materials, including calcium carbonate (CaCO_3;_ 99.5%), calcium hydrogen phosphate dihydrate (CaHPO_4_·2H_2_O; 99.6%), SiC (99.5%), CuO (99.5%), were used to prepare HA and its nanocomposites. However, reagent-graded materials, including sodium bicarbonate (NaHCO_3_; 99.3%), potassium chloride (KCl; 99.2%), calcium chloride (CaCl_2_; 99.4%), dibasic potassium phosphate (K_2_HPO_4_; 99.4%), magnesium chloride (MgCl_2_; 99%), tris-amino-methane ((CH_2_OH)_3_CNH_3_), and hydrochloric acid (HCl; 99.2%) were used to prepare SBF solution for evaluating the bioactivity of the prepared layers in vitro. The densities of CuO (6.31 g/cm^3^), SiC (3.21 g/cm^3^), CaCO_3_ (2.71 g/cm^3^), and CaHPO_4_.2H_2_O (2.31 g/cm^3^). All materials used have been purchased from Sigma-Aldrich.

### Methods

#### Preparation of HA nanopowders

CaCO_3_ and CaHPO_4_.2H_2_O were the essential ingredients used to manufacture HA in this production process^23,24^. Then, using a high-energy ball mill (HEBM) for five hours at 150 rpm and a ball-to-powder ratio (BPR) of 5:1 under dry circumstances at room temperature, 0.386 g of CaHPO_4_.2H_2_O and 4.002 g of CaCO_3_ were weighed and mixed. The following formula provides the fraction of stoichiometry for mixed components:

1 $$6{\text{CaHP}}{{\text{O}}_4}0.2{{\text{H}}_2}{\text{O}}+4{\text{CaC}}{{\text{O}}_3} \to {\text{C}}{{\text{a}}_{10}}{({\text{P}}{{\text{O}}_4})_{6 - x}}{({\text{C}}{{\text{O}}_3})_x}{({\text{OH}})_2}+14{{\text{H}}_2}{\text{O}}+(4 - x){\text{C}}{{\text{O}}_2}$$

With a BPR of 20:1, the mixture powder was processed for ten hours at 450 rpm in a HEBM. The grinding process was split into 60-minute stints with 30-minute breaks to avoid overheating. The fine powders of HA produced were then examined using X-ray diffraction (XRD; Philips PW 1373 X-ray powder diffractometer equipped with CuK–Ni filtered radiation) by pressing on a flat sample holder, ensuring a smooth surface for accurate measurements at room temperature. The powders were dispersed in distilled water using an ultrasonic device, and one or two drops of the powder-containing liquid were placed onto carbon-coated high-resolution transmission electron microscopy (HRTEM) in conjunction with selected area electron diffraction (SAED) JEOL JEM-2100 Japan, operated at an accelerating voltage of 120 kV).

#### Fabrication of FGC layers

**Fig. 1:**
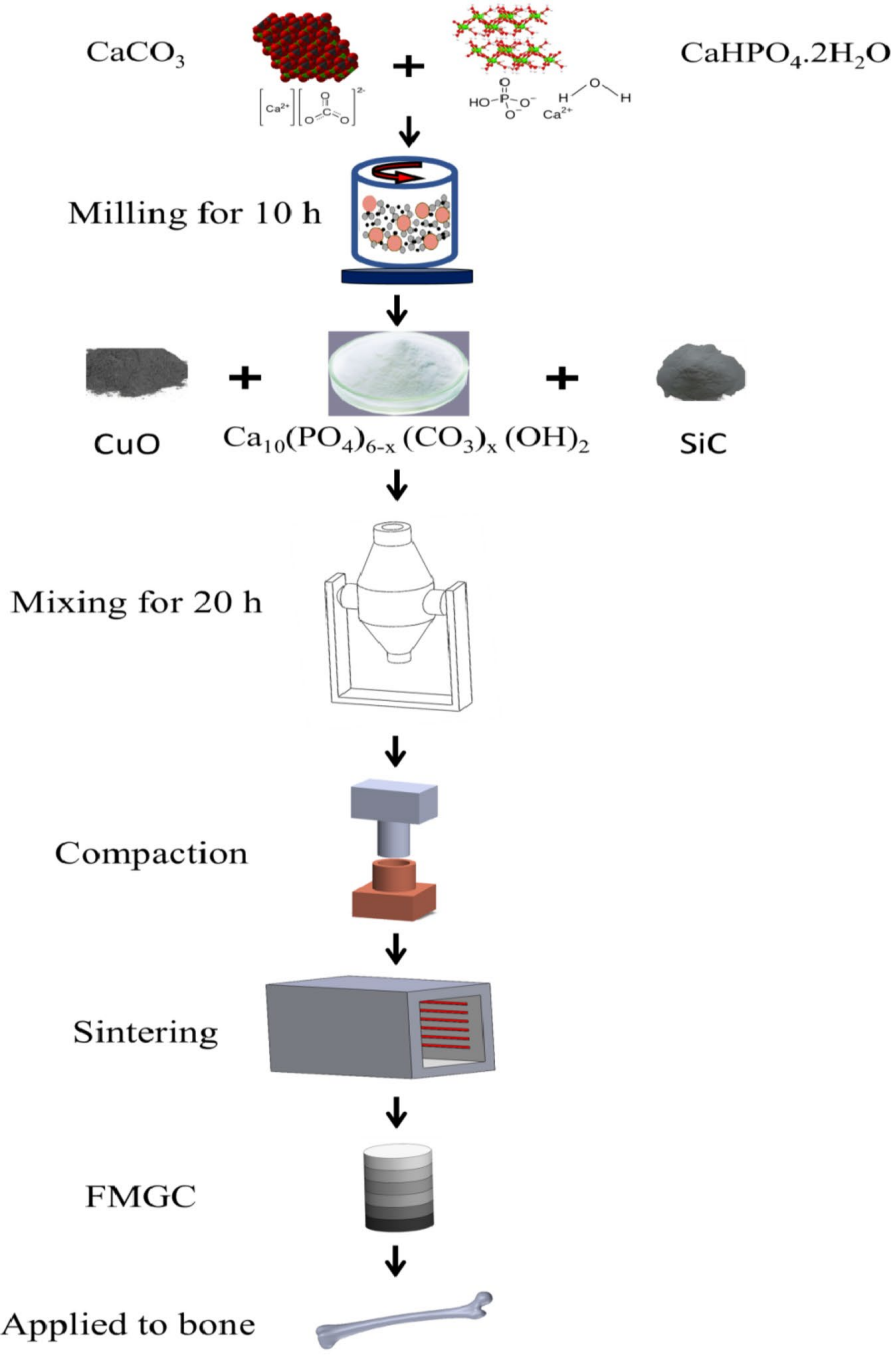
The schematic diagram represents the FGC sample preparation steps.

**Fig. 2:**
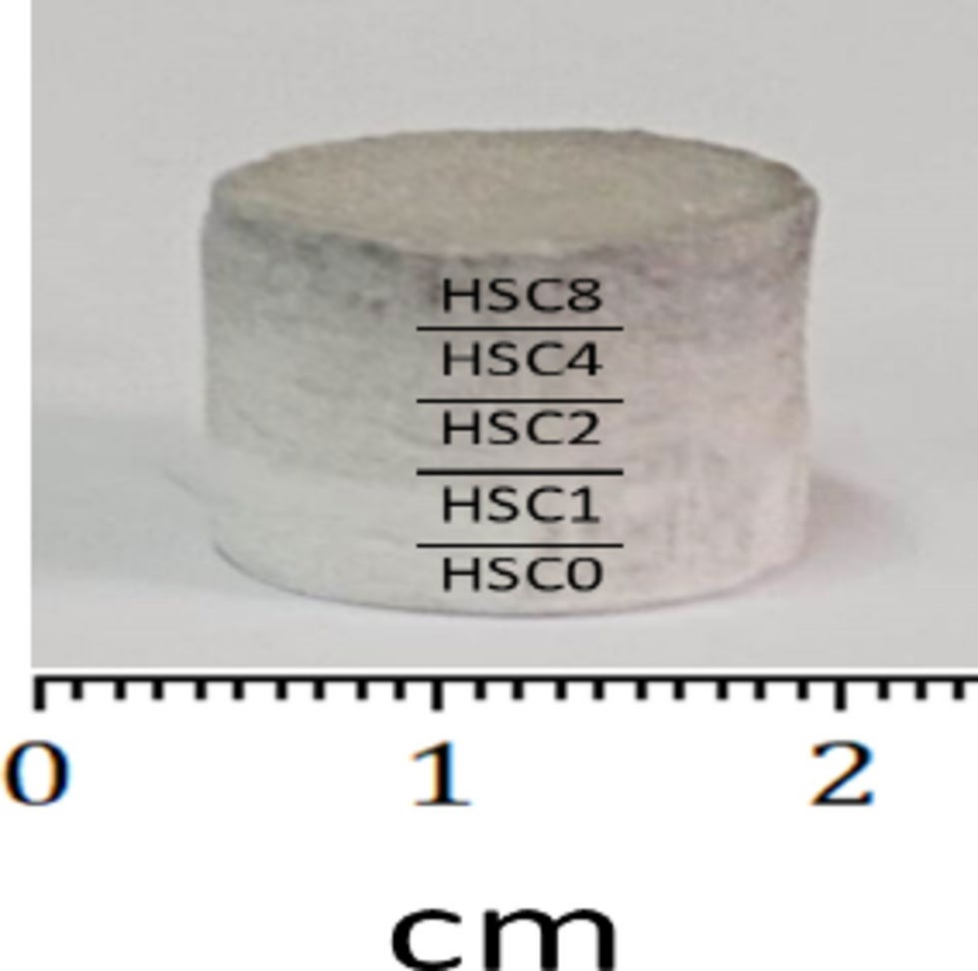
Images showing the different layers forming the FGC sample.

XRD technique and HRTEM-SAED were used to investigate the SiC and CuO nanopowders that had been bought. Five layers, each 4 mm thick, make up the FGC. Along its length, the layers have varying volumetric ratios of HA, CuO, and SiC. The make-up of each layer is shown in Table 1. The BPR was 20:1, and each layer was milled with HEBM for 20 h at 500 rpm. Layer by layer, the FGC was crushed in a hydraulic press for three minutes at 60 MPa, resulting in a thickness of 4 mm and a radius of 7.5 mm. Next, sintering was carried out for two hours at a heating rate of 5 ºC per minute in air at 850 ºC. Figure 1 depicts the procedures involved in FGC preparation, and Fig. 2 shows the pictures that exhibit the various layers.

#### Characterization of sintered FGC layers

The structure of the sintered layers in the FGC was analyzed using the XRD method. Additionally, the microstructure of these layers was examined using field emission scanning electron microscopy coupled with energy dispersive spectroscopy (FESEM-EDS; Quanta FEG250, operated at an accelerating voltage of 30 kV), which offers a magnification range from 10X to 300,000X. Notably, samples were washed with ethyl alcohol before being allowed to dry and then covered with a thin layer of gold to enhance their brightness in preparation for FESEM analysis.

#### Measurement of the different properties of the obtained layers of FGC

##### Physical properties

The physical characteristics of the FGC sample and its layers, such as bulk density and apparent porosity, were assessed in this investigation. Our most recent publication^25^ stated that we determined them using the liquid displacement approach. The following formula was then used to generate these values:

2 $$\:\text{B}\text{u}\text{l}\text{k}\:\text{d}\text{e}\text{n}\text{s}\text{i}\text{t}\text{y}=\frac{{\text{W}}_{\text{d}}}{{\text{W}}_{\text{S}}-{\text{W}}_{\text{i}}}\:\times\:{{\uprho\:}}_{\text{L}}$$

3 $$\:\text{A}\text{p}\text{p}\text{a}\text{r}\text{e}\text{n}\text{t}\:\text{p}\text{o}\text{r}\text{o}\text{s}\text{i}\text{t}\text{y}=\frac{{\text{W}}_{\text{d}}-\:{\text{W}}_{\text{i}}}{{\text{W}}_{\text{S}}-{\text{W}}_{\text{i}}}\:\times\:\:100$$

The fluid density (ρ_l_) was used to calculate the weight of a sample immersed in liquid, represented by the symbol Wi. Additionally, the weights of the dry sample (W_d_) and the saturated sample in the air (W_s_) were considered. To guarantee repeatability, it should be mentioned that these measurements were carried out five times.

##### Mechanical properties

Before and after the sintered layers were soaked in the SBF solution for 14 days, measurements were made of their mechanical properties, such as microhardness, ultimate strength, and a set of elastic moduli. A Vickers hardness machine (model: Shimadzu Corporation hardness tester) was used to measure the microhardness of the FGC layers following^26^ after polished samples were subjected to 1.961 N for ten seconds at room temperature. Determine the hardness by taking the ratio of the indenter’s hierarchical contact area to the applied load. One way to represent Eq. (4) is as follows:

4 $$\:\text{H}\text{v}=\:1.854\frac{\:\text{P}}{{\text{D}}^{2}}$$

P is the applied indentation load, and D is the measured indentation diagonal. The Vickers microhardness profile was evaluated for a longitudinal section of the FGC sample to show the microhardness profile. The reading was taken for every 1 mm length of the sample.

The mechanical tester (Instron, Darmstadt, Germany) was used to evaluate the sintered samples’ ultimate strength (σ). One millimeter per minute was the continuous pace at which the compression tests were conducted. σ was measured using the following equation:

5 $$\:{\upsigma\:}=\:\frac{{\text{F}}_{\text{f}}}{\text{A}}$$

F_f_ represents the load at fracture, and A represents the disc surface area.

A minimum of five indentations were measured for each specimen to generate every data point. While the ASTM E9 standard was used to evaluate the ultimate strength of each specimen, the pulse-echo technique was employed to measure ultrasonic wave velocities (longitudinal and shear) propagating through the samples at ambient temperature. This was done using MATEC Model MBS8000 DSP (ultrasonic digital signal processing) equipment with a 5 MHz resonator. According to^27–30^, the longitudinal (V_L_^2^) and shear (V_S_^2^) ultrasonic velocities were used to calculate the values of Lame’s constants, namely λ and µ.

6 $$\:{\uplambda\:}={\uprho\:}({V}_{L}^{2}-2{\text{V}}_{S}^{2})$$

7 $$\:{\upmu\:}\:={\uprho\:}{V}_{S}^{2}\:$$

8 $$\:L=\lambda\:+2\mu\:$$

9 $$\:G=\mu\:$$

10 $$\:E=\mu\:\frac{3\lambda\:+2\mu\:}{\lambda\:+\mu\:}$$

11 $$\:B=\lambda\:+\frac{2}{3}\mu\:$$

where *ρ* is the measured density of samples, L is the longitudinal modulus, G is the shear modulus, E is the modulus of elasticity, and B is the bulk modulus.

An average of five readings along the specimens’ cross-sectional surfaces was used to evaluate the mechanical properties.

##### Electrical and dielectric properties

The broadband dielectric spectroscopy method, which uses an increased-resolution ALPHA analyzer with a (Model: Novo control, Montabaur, Germany), was used to do the dielectric research in the frequency range of 10^− 1^ to 10^7^ Hz. Two 10 mm diameter, gold-plated stainless steel electrodes were positioned between sintered samples before and after they soaked in SBF solution, creating a parallel plate capacitor arrangement. Interestingly, these measurements were taken five times.

##### Wear resistance

To conduct the wear test, we employed a digital balance with a precision of 0.0001 to weigh and measure the samples precisely. The test was conducted using pin-on-disk tester equipment. The samples underwent a meticulous polishing process utilizing grinding papers with varying grades, ranging from 600 to 4000. The samples were standardized to possess identical dimensions, measuring 4 mm in thickness and 7.5 mm in radius. Three different weights-exactly 10, 20, and 40 N-were employed in the experiment. RS steel was used to construct the tribometer disc and pin. To find out how much wear the sample had, a wear test was conducted in dry circumstances, measuring each applied force at a distance of 480 m, speed = 0.8 m/sec., and time = 600 s. Formulae were used to determine the sintered samples’ weight loss (ΔW) and wear rate (W)^31,32^.

12 $$\:\varDelta\:W=\:{W}_{before}-{\text{W}}_{after}$$

13 $$\:\:\text{W}\:\left(\frac{{\text{m}\text{m}}^{3}}{\text{m}}\right)=\frac{\varDelta\:W}{\text{D}\:\text{x}\:{\uprho\:}}$$

The specific wear rate (k) was also calculated according to^33^.

14 $$\:\text{K}\:\left(\frac{{\text{m}\text{m}}^{3}}{\text{N}\text{m}}\right)=\frac{\varDelta\:W\:}{\text{D}\text{x}\:{\uprho\:}\text{x}\:\text{P}}\:$$

Where ΔW is the weight loss (mg), W_before_ is the weight of the sample before the wear test (mg), W_after_ is the weight of the sample after the wear test (mg), ρ is the bulk density (g/mm^3^), D (m) is the sliding distance (m) and P is applied load (N).

##### Biological properties

*Bioactivivty*. In this work, the samples’ bioactivity was evaluated in vitro using SBF solution, which is similar to human blood plasma and was prepared according to the Kokubo et al.^34,35^. recipe, keeping in mind that the sample-to-solution ratio was 0.01 g/ml^36,37^. The composition of the prepared SBF solution is listed in Table 2.

Each sintered layer was submerged in the SBF solution at 37 ± 0.5 °C for fourteen days, or body temperature on average. The samples were removed after immersion and washed in distilled water before and after being dried for about an hour at 80 °C in an oven. The coating HA layer that formed on the sample surfaces was then seen using FESEM pictures. One of the best indicators of a sample’s bioactivity is the precipitation of an apatite layer on its surface.

*Antibacterial investigation*. The antibacterial activity of the FGC sample’s generated layers against two common species of Gram-positive and Gram-negative bacteria, Staphylococcus aureus (*S. aureus;* ATCC 6538) and Escherichia coli (*E. coli;* ATCC 25922) bacteria was evaluated in the current study using the disc-diffusion method. The test was conducted by incubating the plates at 37 ± 0.1 °C for 24 h to encourage bacterial growth, using Ofloxacin (Oxoid) as a reference antibiotic. The millimeter inhibitory zones were measured using a ruler after incubation.

*Analysis of the cytotoxic potential of materials made from a human osteosarcoma cell line*. The cytotoxic effects of the specimens were evaluated using the human osteosarcoma Saos-2 cell line (ATCC, USA) at the Bioassay–Cell Culture Laboratory of the National Research Centre in Egypt. Using a microplate multi-well reader (Bio-Rad Laboratories Inc., model 3350, Hercules, California, USA) and a reference wavelength of 620 nm, the absorbance was measured. The statistical significance between the specimens and the negative control was investigated. IC50 and IC90 probit analyses were performed using the SPSS11 program. The percentage change in possibility was estimated using the following formula:

15 $$\:\left(\frac{\text{R}\text{e}\text{a}\text{d}\text{i}\text{n}\text{g}\:\text{o}\text{f}\:\text{e}\text{x}\text{t}\text{r}\text{a}\text{c}\text{t}}{\text{R}\text{e}\text{a}\text{d}\text{i}\text{n}\text{g}\:\text{o}\text{f}\:\text{n}\text{e}\text{g}\text{a}\text{t}\text{i}\text{v}\text{e}\:\text{c}\text{o}\text{n}\text{t}\text{r}\text{o}\text{l}}-1\:\right)\text{x}\:100$$

## Results and discussion

### Characterization of the starting materials

**Fig. 3:**
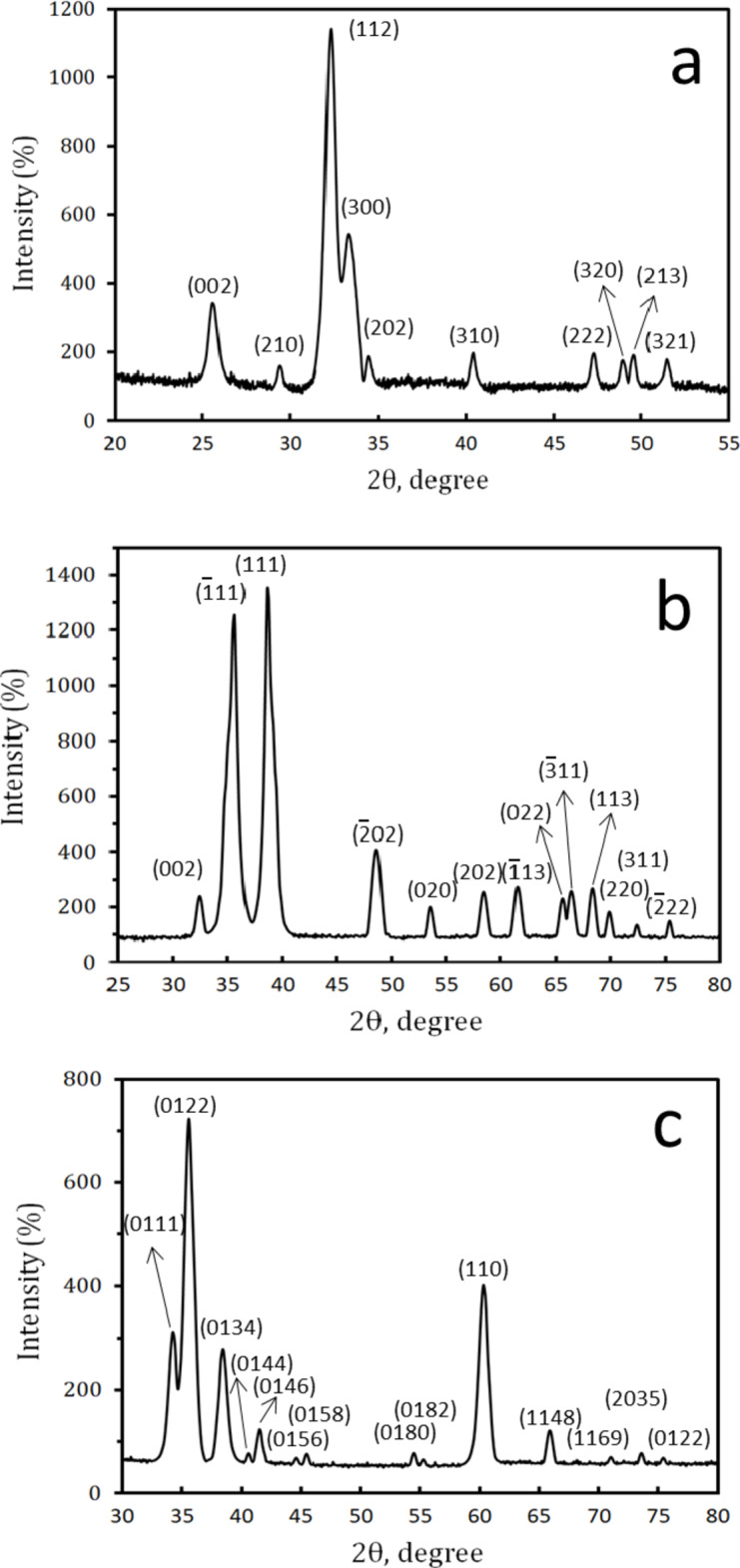
XRD patterns of the starting materials, namely (a) HA, (b) CuO, and (c) SiC powders.

**Fig. 4:**
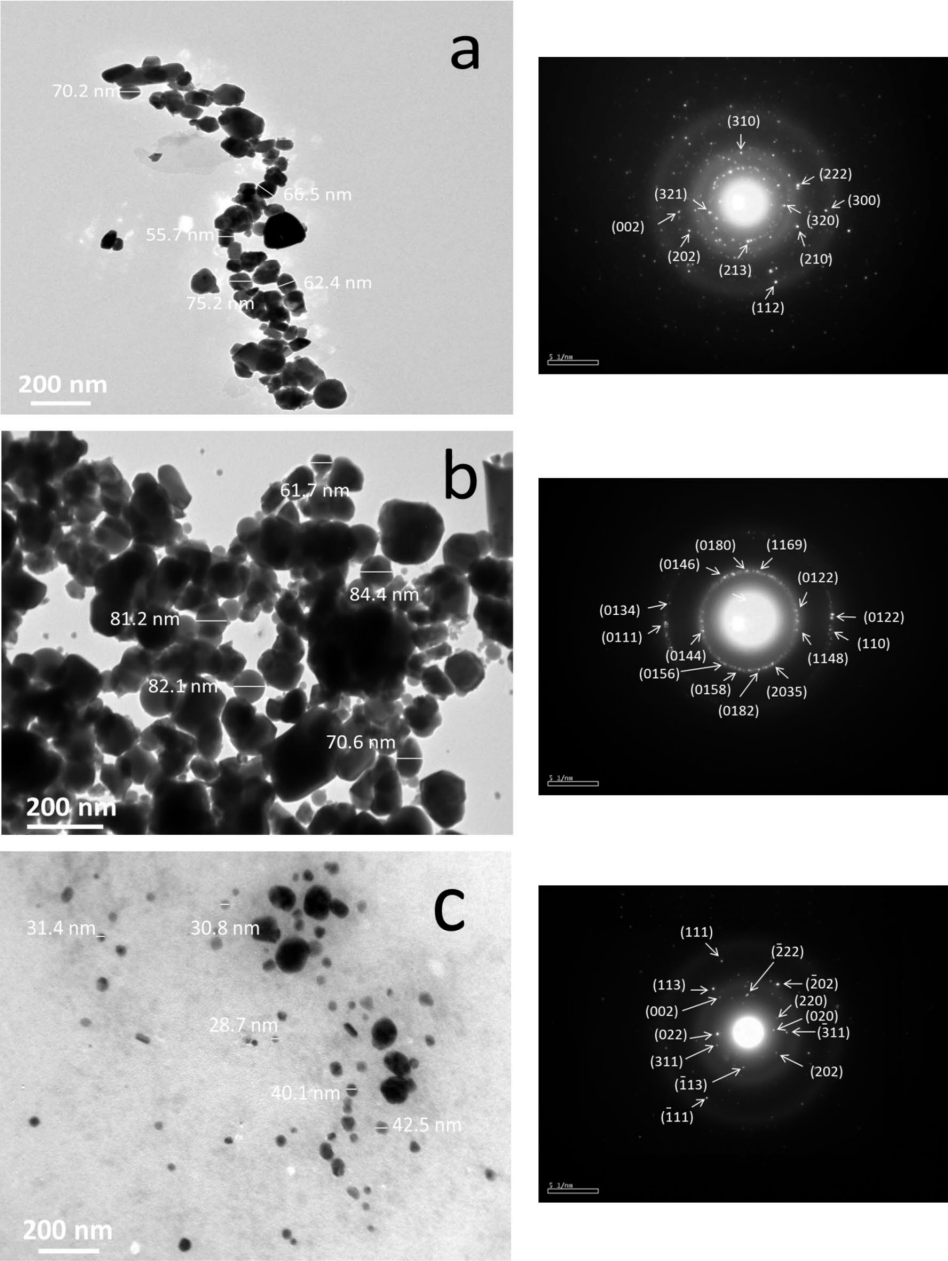
HRTEM images coupled with SAED patterns of raw materials, i.e., (a) HA, (b) CuO, and (c) SiC nanopowders.

The phase composition, particle sizes, and crystallinity of HA, CuO, and SiC were determined using XRD and HRTEM-SAED methods, as seen in Figs. 3(a–c) and 4(a-c), respectively. The distinct and well-defined XRD peaks at 2θ = 32.19°, 33.34°, 25.74°, and 40.43°, which correspond to (1 1 1), (3 0 0), (0 0 2) and (3 1 0), respectively, according to (ICCD file card: 19–0272), make it evident from Fig. 3a that HA was synthesized efficiently. At 2θ = 38.75°, 35.55°, 38.92°, 35.46°, 48.70°, and 61.55°, the distinctive XRD peaks of CuO, Fig. 3b, were identified using (ICCD file card: 89-5899). These correspond to (1 1 1), (1̅ 1 1), (2 0 0), (0 0 2), (2̅ 0 2), and (1̅ 1 3), respectively. The development of the typical XRD peaks recognized according to (ICCD file card: 89-2225) at 2θ = 35.60°, 60.13°, 34.14°, and 38.31°, which correspond to (0 6 9), (1 1 0), (1 1 1), (3 1 0) and (1 3 5), respectively, indicated the existence of SiC, as shown in Fig. 3c. For HA, CuO, and SiC, all diffraction peaks are broader than the typical pattern, suggesting that the materials used in this investigation are nanostructured. Because nanostructured materials interact better with proteins and bone cells, biomedical researchers stand to gain a great deal from their development^38^.

With average particle sizes of 66, 76, and 34.7 nm, respectively, the HA, CuO, and SiC powder images generated in Fig. 4(a-c) are easily recognizable as composed of spherical particles in the nanoscale range. There was significant aggregation in both HA and CuO. Conversely, because of its great strength, this aggregation is far less in SiC than in Ha and CuO. Furthermore, based on the d-spacing ICCD file cards mentioned above (1 1 1), (3 0 0), (1̅ 1 1), (0 6 9), and (1 1 0), respectively, the SAED patterns showed the existence of polycrystalline diffraction rings in the *h k l* planes of HA, CuO, and SiC.

### Characterization of the sintered FGC layers

#### Phase composition

**Fig. 5:**
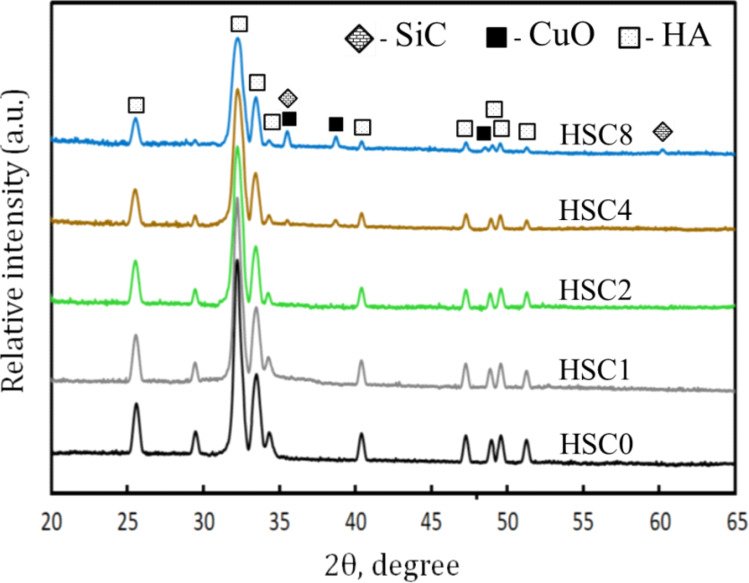
XRD patterns of all FGCs layers sintered at 850 °C for two hours.

In this study, XRD measurements were made for two reasons. Before beginning the grinding process, ensure that the materials are free of contamination from the grinding jars and balls. Second, confirm that the sintering procedure did not cause any reactions and that the produced HA did not break down when exposed to the sintering temperature. In this context, the XRD patterns of every sample sintered at 850 °C were captured and shown in Fig. 5. This figure displays the XRD peaks of HA, CuO, and SiC, as explained in Sect. 3.1. The sharpness of the distinctive XRD peaks shows that the sintering temperature has a beneficial effect on the materials’ crystallinity compared to the XRD patterns produced before and after sintering. As anticipated, there is no indication of any additional phases or contaminants, and the peaks associated with HA show a consistent decrease in intensity as the CuO and SiC contents rise.

#### Surface morphology

**Fig. 6:**
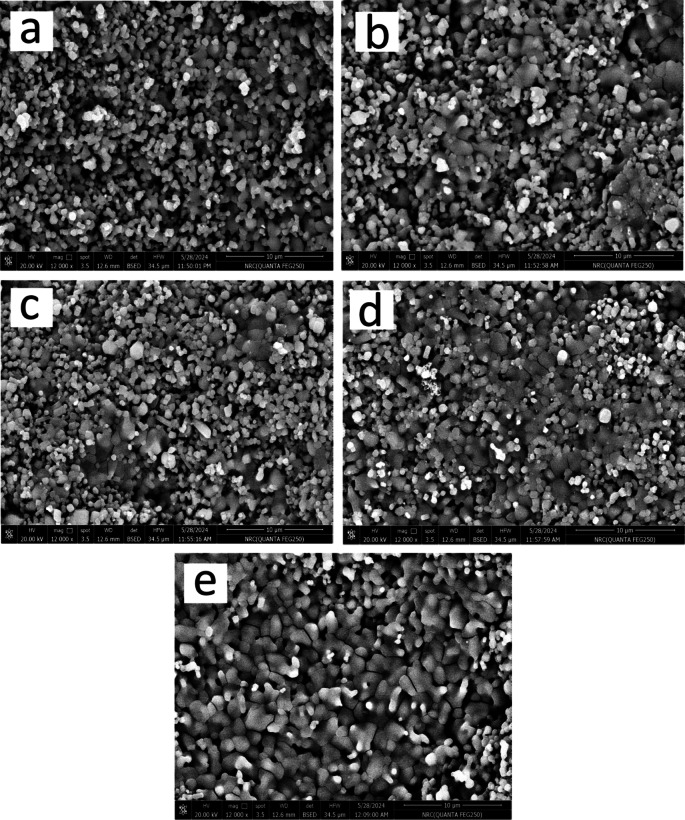
FESEM micrographs of (a) HSC0, (b) HSC1, (c) HSC2, (d) HSC4, and (e) HSC8 layers sintered at 850 °C for two hours.

As shown in Fig. 6(a-e), FESEM was used to assess the microstructure of every sintered sample. The notable reduction in porosity and the increase in grain size show that the chosen sintering temperature produced excellent densification properties for all sintered layers. However, it was unable to achieve an utterly condensed design. As long as the mechanical characteristics of the implants are not adversely affected, this discovery is advantageous for a wide range of biomedical applications. Porosity is thus necessary to improve the integration of implants with bone, encourage cell division, and facilitate the development of new blood vessels. Simply put, the presence of live bone cells and the extracellular matrix in an implant with limited porosity restricts the formation of blood vessels around it^39^.

It is logical to believe that a gradual increase in the content of CuO would marginally enhance densification, as the melting temperatures of HA, CuO, and SiC are 1670, 1326, and 2730 °C, respectively. This result is consistent with research presented in^40,41^, which demonstrated how CuO can improve the compaction of various ceramic materials. We will now go into more detail about the condensation process’ mechanics to make things more straightforward for the reader.

The selection of the sintering temperature during the three phases of the sintering process determines the quality of the densification. Contact establishment is improved by powder compaction. Furthermore, the formation of interwoven “necks” connecting the particles shows a strong association between them. When the temperature during sintering rises to two-thirds of the melting point, these necks are formed. Any residual porosity is effectively sealed up when the particles eventually become so fully bound that it is hard to tell them apart. Applying the concepts above to the obtained FESEM images, it is clear that the HA particles in Fig. 6a are small and visible as distinct entities, suggesting that they do not ordinarily diffuse. The existence of porosity in the material under study is due to the observed decrease in diffusion. Adding one vol% of CuO to the second sample, HSC1, as shown in Fig. 6b, leads to a bit of improvement in densification. The observation of increasing particle sizes, where some particles are close together and cannot be distinguished separately, leads to this conclusion. As shown in Fig. 6c, increasing CuO content in the HSC2 sample intensifies the process above and significantly reduces porosity. In the HSC4 and HSC8 samples, the influence of these effects is more apparent.

**Fig. 7:**
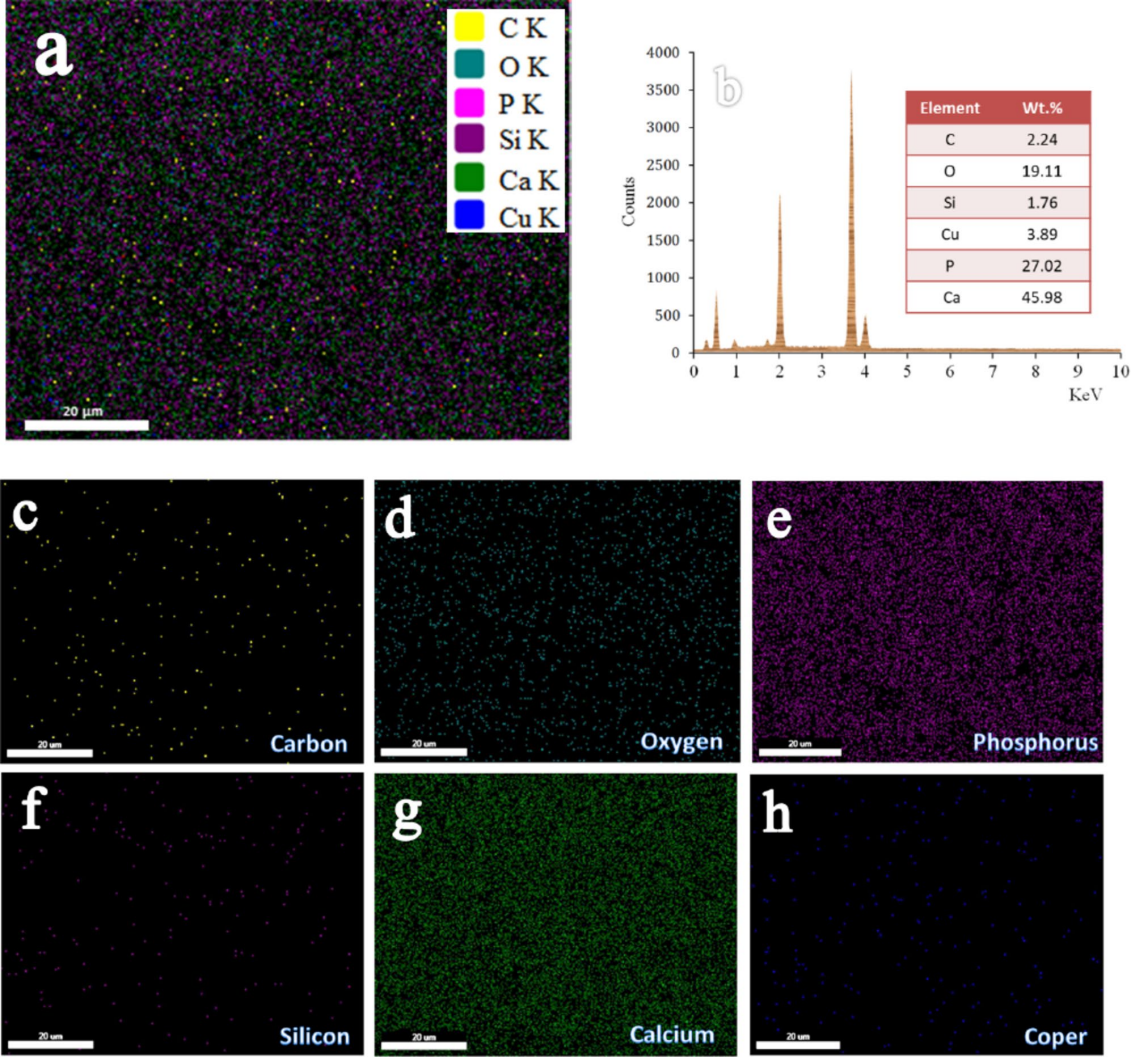
(a) EDX mapping of all constituents of the HSC8 sample, (b) EDX spectrum of HSC8 sample and elemental mapping of each constituent in the HSC8 sample, i.e., (c) carbon, (d) oxygen, (e) phosphorous, (f) silicon, (g) calcium, and (h) copper.

Figure 7(a-h) displays the EDX mapping of all components in the HSC8 sample, including the EDX spectrum and a thorough elemental mapping for each element. In addition to verifying the findings in Sect. 3.2.1, which showed no extra components beyond this sample, this figure shows the homogenous distribution of every aspect of the HSC8 sample.

### Measurement of the different properties of the obtained FGC

#### Physical properties

**Fig. 8:**
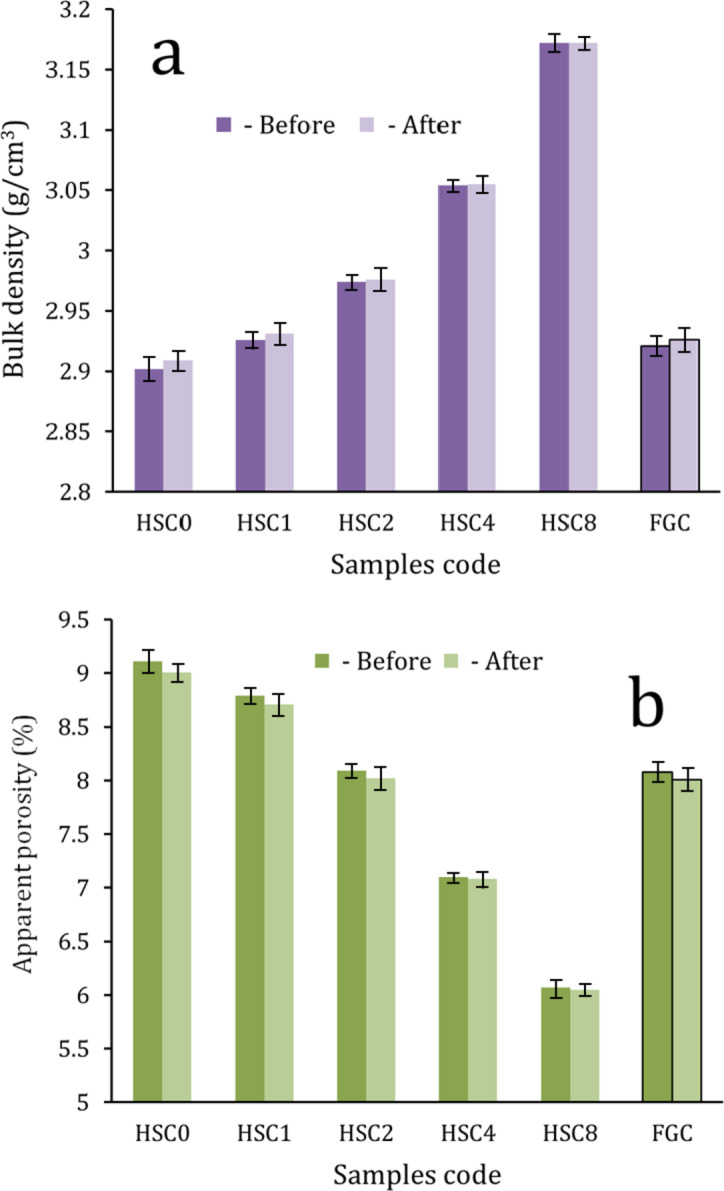
(a) Bulk density and (b) apparent porosity of the FGC sample and its different layers before and after soaking in the SBF solution for fourteen days.

The densification of ceramic materials is substantially influenced by several factors, including sintering temperature, environment, and initial powder particle size^42^. Figure 8(a, b) illustrates the bulk density and apparent porosity of the FGC sample and its various sintered layers, respectively. The data indicate that the bulk density of the generated layers grows exponentially with the rising concentration of CuO. The increase in bulk density is due to the substitution of the lighter material, HA (3.15 g/cm^3^), with the denser element, CuO (6.31 g/cm^3^). The bulk density significantly rose by 1.03%, 2.06%, 6.89%, and 9.60% due to the continuous rise in the concentrations of SiC and CuO. This finding is significant since the materials are specifically designed for potential use in orthopedic applications.

Consequently, developing nanocomposites with appropriate properties is essential while reducing the increase in bulk density. SiC was chosen as an alternative for these nanocomposites due to its remarkable mechanical characteristics and low density. These results correspond with those presented in Sect. 3.2.2. The porosity has a distinct pattern compared to the density values. The value decreases markedly with increased CuO concentration, even when SiC ratios rise to 4 vol%.

The porosity values reported for the FGC sample and all sintered layers are 9.2%, 8.7%, 8.2%, 7.2%, 6.2%, and 8.2%. The obtained findings may be clarified by examining two factors. A significant finding is that the melting points of HA and CuO are around half that of SiC. This attribute is crucial as it facilitates the closing of pores in the sintered specimens. Moreover, nanopowders possess the capability to close pores^42^. This result demonstrates that layers may be fabricated with high density without the disintegration of CHA due to the employed sintering temperature, presenting a significant challenge to researchers. The porosity values measured for sintered layers are similar to those of compact bone, varying between 5% and 13%^43^.

#### Tribo-mechanical properties

**Fig. 9:**
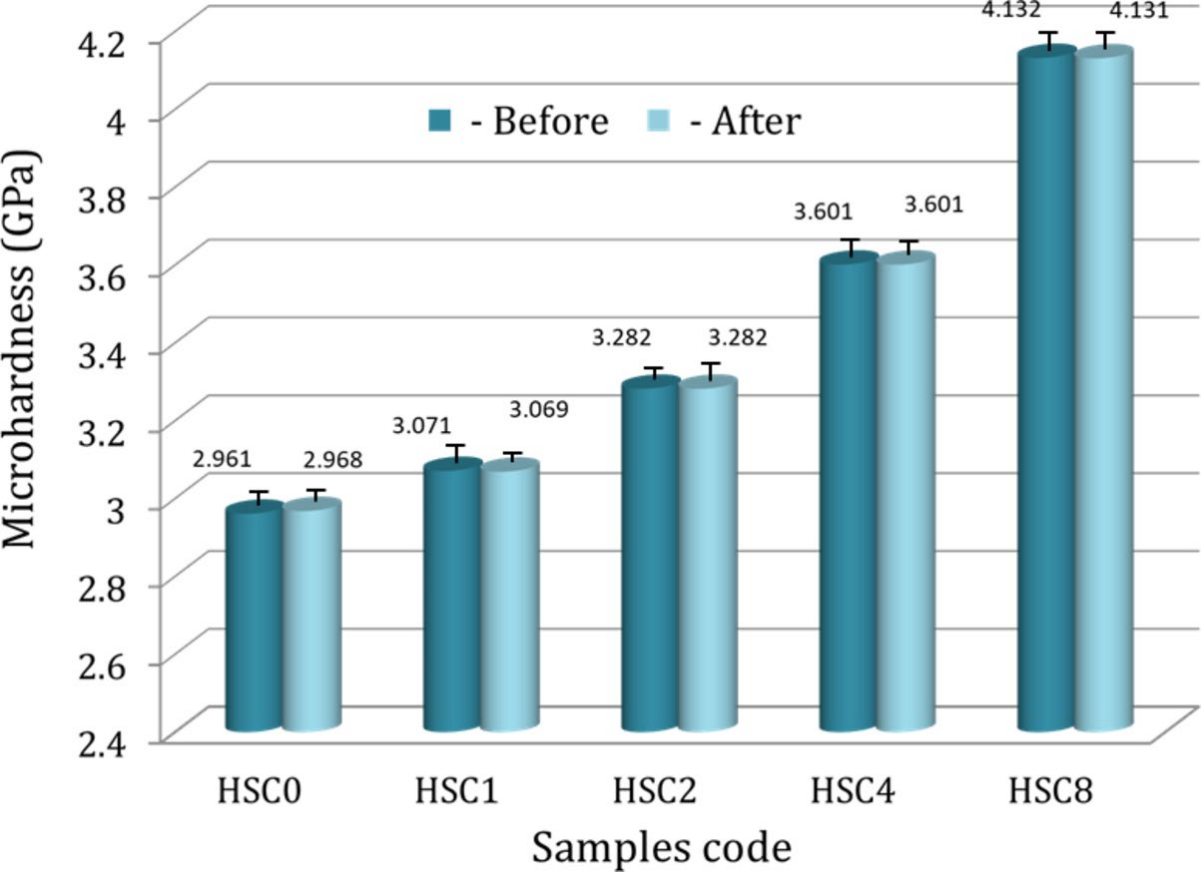
The microhardness of all FGC layers sintered at 850 ℃ for two hours before and after soaking in the SBF solution for fourteen days.

**Fig. 10:**
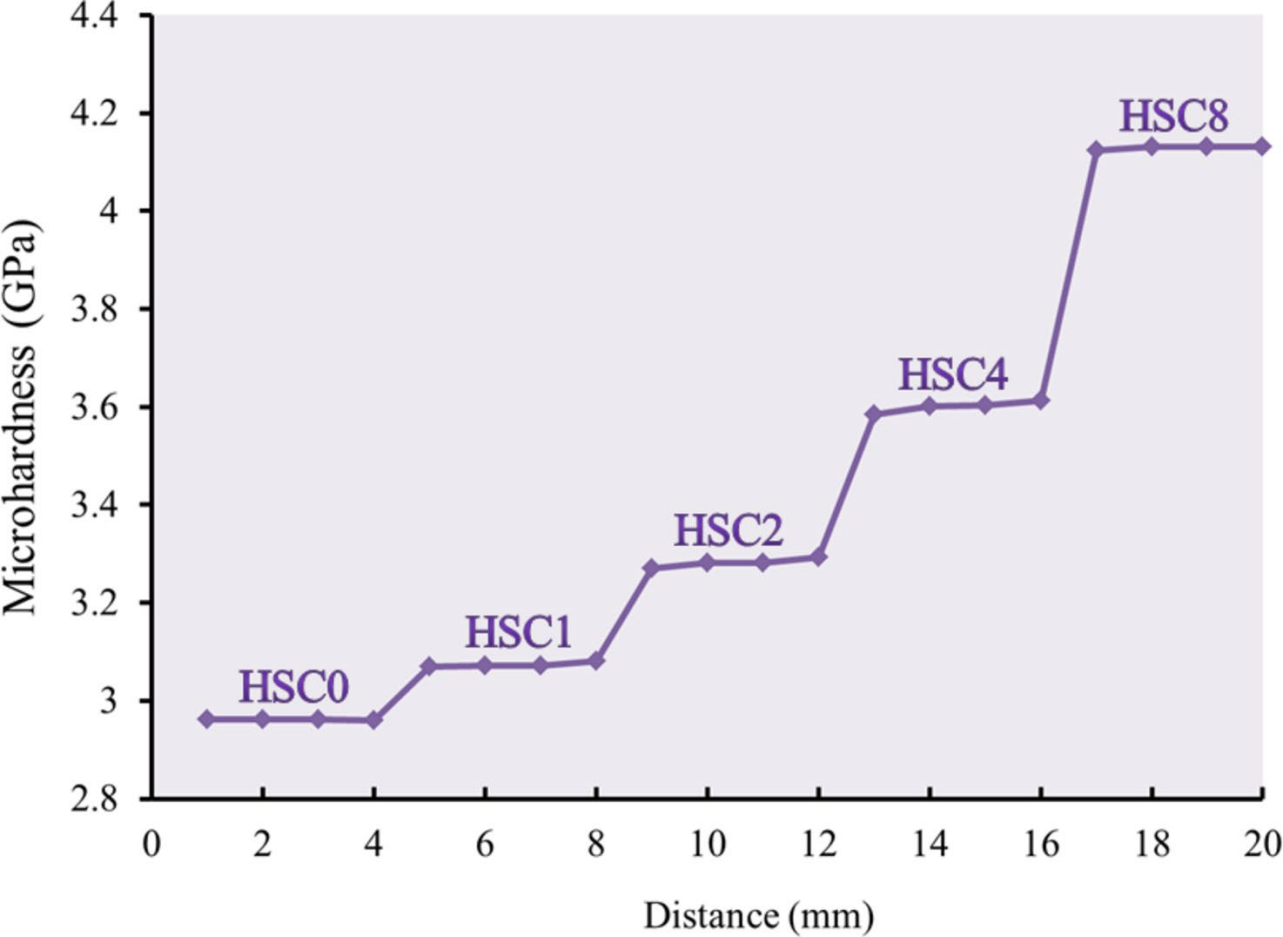
Microhardness profile of a longitudinal section of the FGC sample measured per millimeter along the length of the sample.

**Fig. 11:**
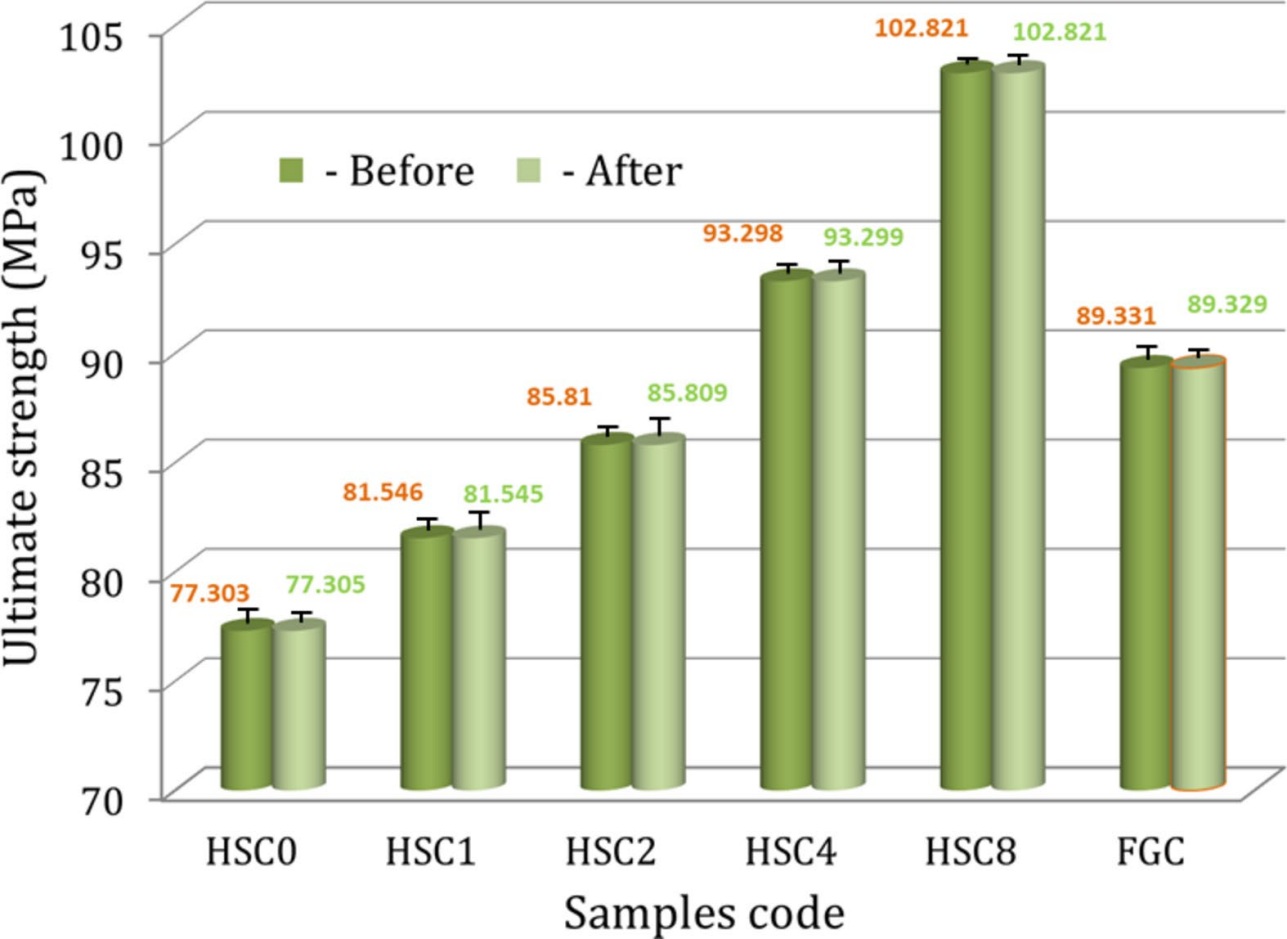
The ultimate strength of the FGC sample and its layers sintered at 850 ℃ for two hours before and after soaking in the SBF solution for fourteen days.

**Fig. 12:**
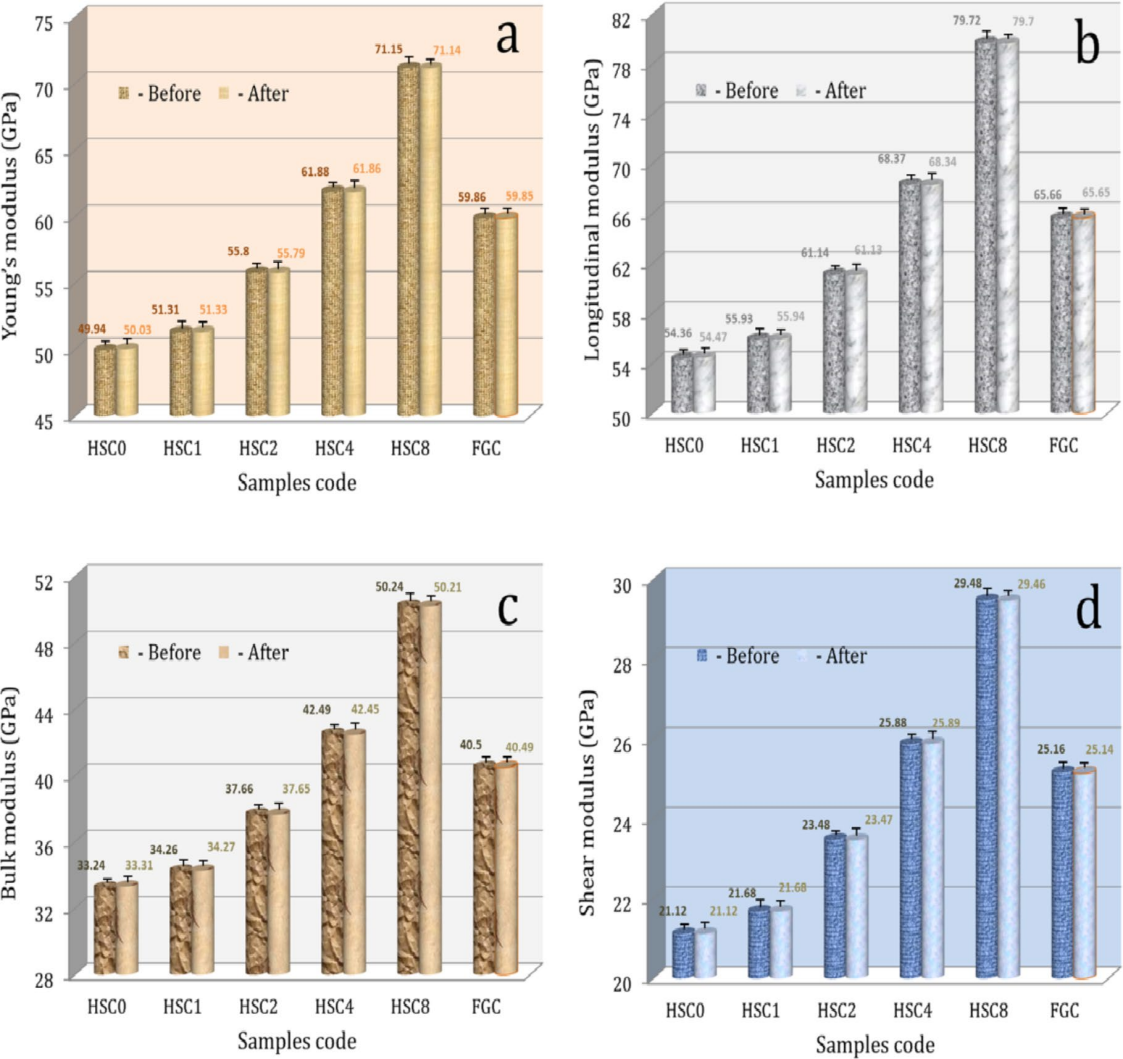
(a) Young’s modulus, (b) longitudinal modulus, (c) bulk modulus, and (d) shear modulus of the FGC sample and its different layers before and after soaking in SBF solution for fourteen days.

Microhardness, microhardness profiles, ultimate strength, and ultrasonic properties, such as Young’s modulus, longitudinal modulus, bulk modulus, and shear modulus for the FGC sample and its layers, are shown in Figs. 9, 10, 11 and 12. These figures show that all measured mechanical properties increased with increasing SiC and CuO contents. In other words, the mechanical properties increase in an ascending manner in the order of HSC0, HSC1, HSC2, HSC4, and HSC8. As expected, the entire FGC sample showed a value approximately in the middle. With the growth of SiC and CuO, the maximum values have reached 4.13 GPa, 102.8 MPa, 71.15 GPa, 79.72 GPa, 50.24 GPa, and 29.48 GPa, respectively, whereas CuO and SiC-free layer values are 2.96 GPa, 77.30 MPa, 49.94 GPa, 54.36 GPa, 32.24 GPa, and 21.12 GPa, respectively. Interestingly, the microhardness profile shows the same behavior as the microhardness value for the examined FGC layers. The results are good densification behavior and improved SiC and CuO mechanical characteristics.

Compared with the results described in previous literature regarding the improvement of HA by adding different types of ceramics or polymers, the mechanical properties of these samples at this relatively lower sintering temperature, i.e., 850 °C, are excellent. For example, Shahbaz et al.^44^ added different amounts of magnesium fluoride up to 10 wt% to HA using the PM technique followed by a sintering process at three different temperatures, i.e., 900, 1000, and 1100 °C, and they observed that the highest microhardness value was 2.2 GPa. Abere et al.^45^ studied the effect of adding 30 vol% of chitosan on the mechanical properties of HA. They found that the values of strength and microhardness were 10.12 MPa and 4.11 GPa, respectively. It should be noted that the ultimate strength of HSC4 and HSC8 samples is quite similar to that of cortical bone (100–150 MPa). This implies that the stress-shielding action would not harm the surrounding bone if these samples were transplanted into human bone. It should be noted that the stress-shielding effect is particularly harmful following Wolff’s law^46^, which results in severe bone degeneration since there are insufficient stimuli for the continuous remodeling process.

**Fig. 13:**
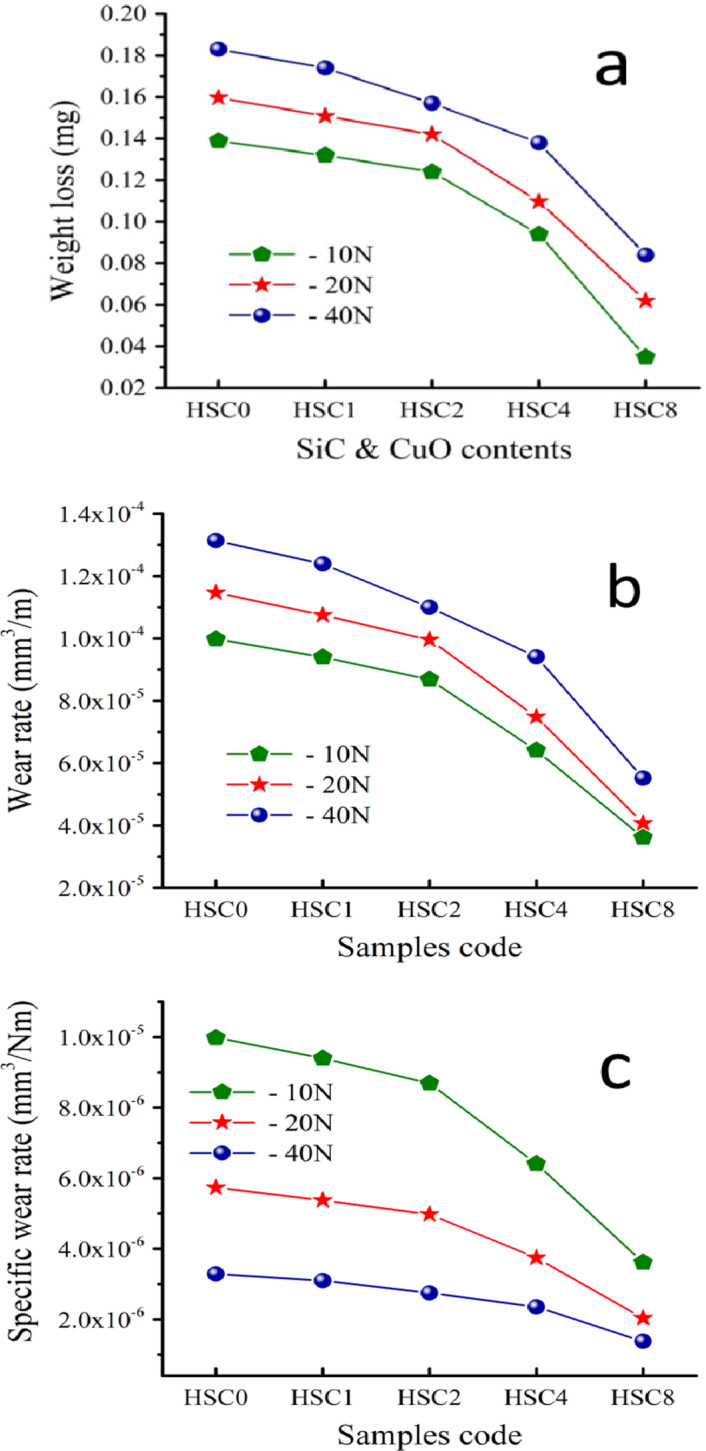
(a) Weight loss, (b) wear rate, and (c) specific wear rate of all samples at different loads, namely 10, 20, and 40 N.

Many people think bodily fluids are the only lubricant surrounding an implant in a human bone. Therefore, a lot of wear debris from inadequate lubrication might cause osteoclastic cells to break down the bone and release the implants. Studying the tribological characteristics of biomaterials for possible use in the bone healing process is essential in light of this critical finding^47^. Accordingly, all samples created at different loads had their weight loss, wear rate, and specific wear rate examined, as shown in Fig. 13(a-c). Wear behavior was improved due to the increases in SiC and CuO concentration. For instance, when load = 10 N was given to the specimens under study, the weight loss decreased by 7.14%, 14.28%, 33.57%, and 75% by increasing the SiC and CuO concentration.

Nevertheless, raising the load to 40 N decreased the weight loss by 2.77%, 12.22%, 22.22%, and 54.44% for the exact volume percentages of CuO and SiC. In addition, the wear rate decreased by 4.34%, 8.69%, 30.43%, and 73.91% by successively increasing the SiC and CuO content at a load of 10 N, and when the load was increased to 40 N, the wear rate decreased by 6.66%, 13.33%, 23.33%, and 53.33%, respectively. This encouraging result can be attributed to several factors. First, using nanomaterials in preparation for FGCs has the great advantage of allowing the particles to be tightly bound to each other. Second, as discussed in Sect. 3.3.1, the sintered samples achieved adequate densification, effectively reducing the samples’ weight loss and wear rate. Finally, hard elements such as CuO and SiC in the sintered samples reduced the wear behavior. The mechanism of this improvement can be explained as follows:

The HA nanoparticles are compressed when the average load (10 N) is applied in the absence of CuO and SiC, and the gaps between the particles become narrower. Compared to HA/SiC/CuO nanocomposite, the stylus can easily separate the nanoparticles of the HSC0 sample and penetrate to greater depths because HA has a low hardness and elasticity modulus. This allows for more extensive stylus impressions because the nano − and micro − cracks initiate and propagate through the substrate with less resistance. Due to its significant role in improving the diffusion process, CuO may fill the gaps between the particles, improving wear resistance and inhibiting nanoparticle separation. Similarly, hard ceramic materials were necessary to improve HA tribological measurements^48–51^.

#### Electrical and dielectric properties

**Fig. 14:**
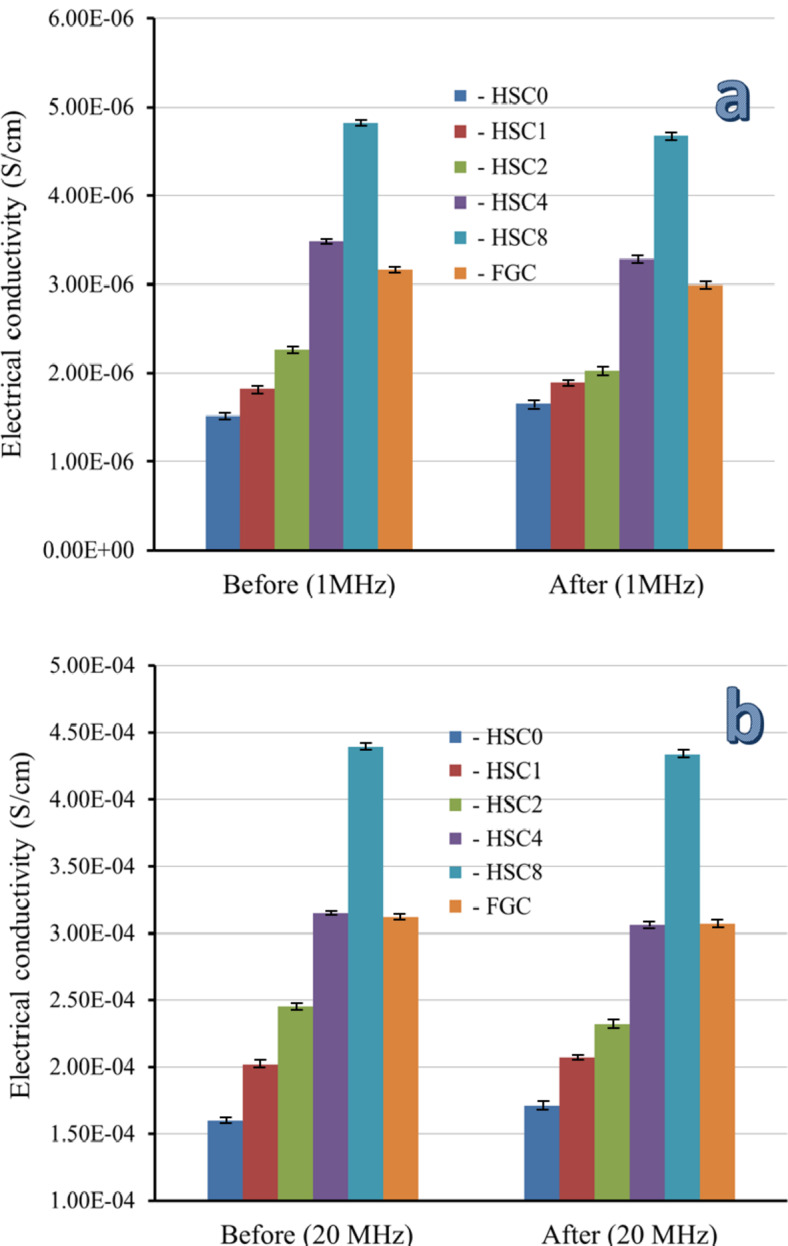
AC electrical conductivity of the FGC sample and its sintered different layers at (a) 1 MHz and (b) 20 MHz before and after soaking in the SBF solution for 14 days.

**Fig. 15:**
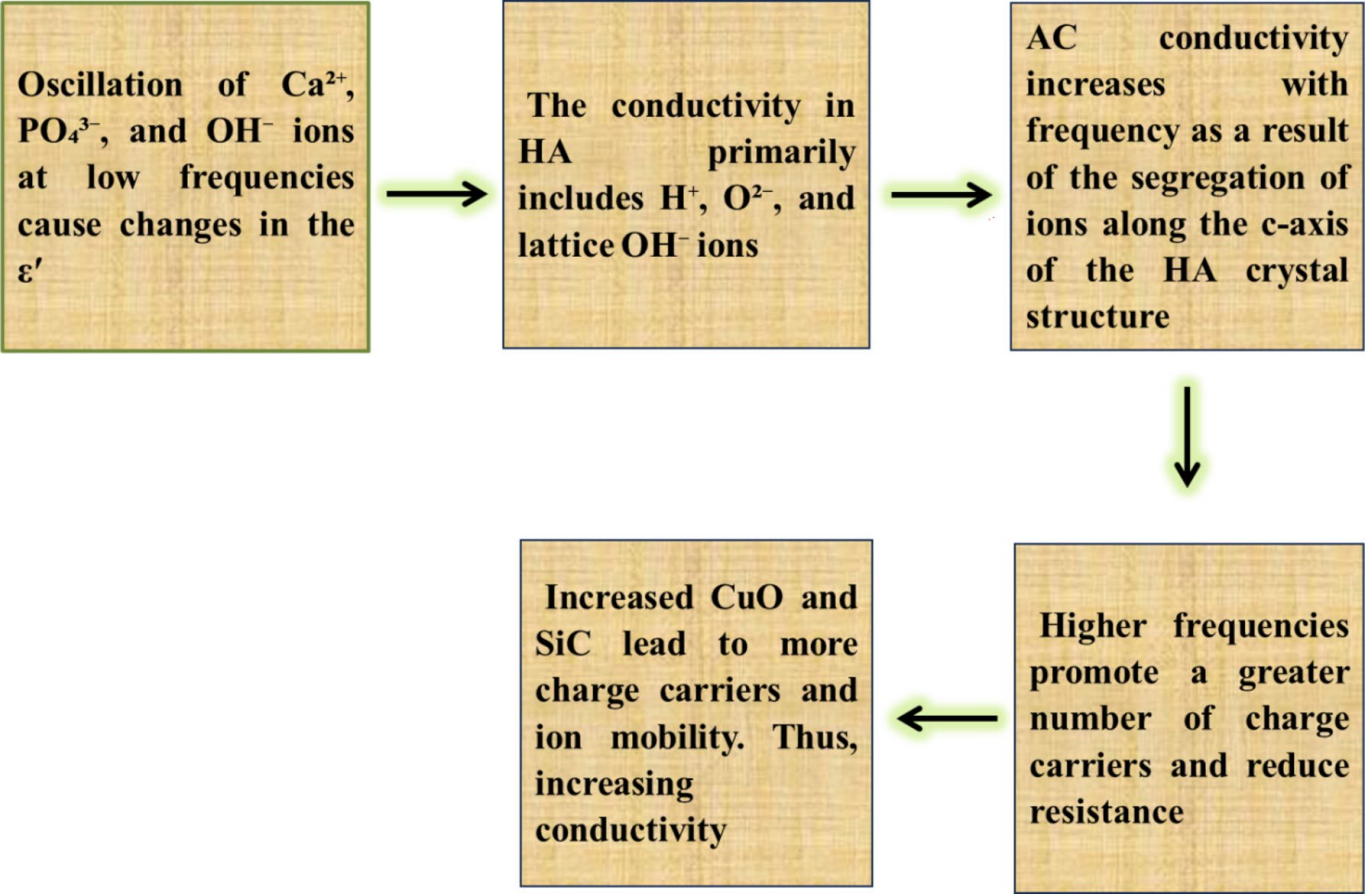
A schematic diagram illustrating the conduction mechanism.

**Fig. 16:**
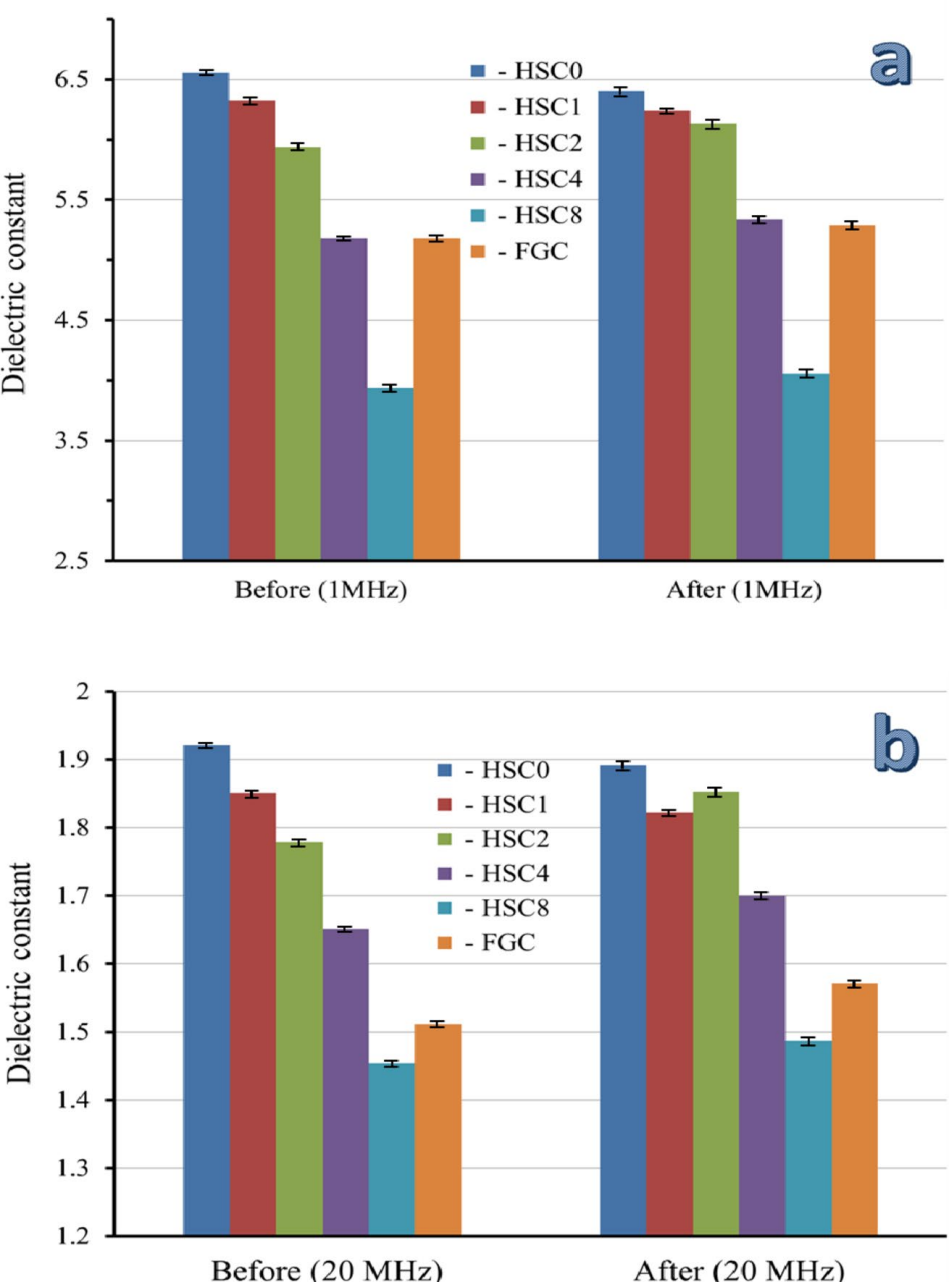
Dielectric constant of the FGC sample and its sintered different layers at (a) 1 MHz and (b) 20 MHz before and after soaking in the SBF solution for fourteen days.

**Fig. 17:**
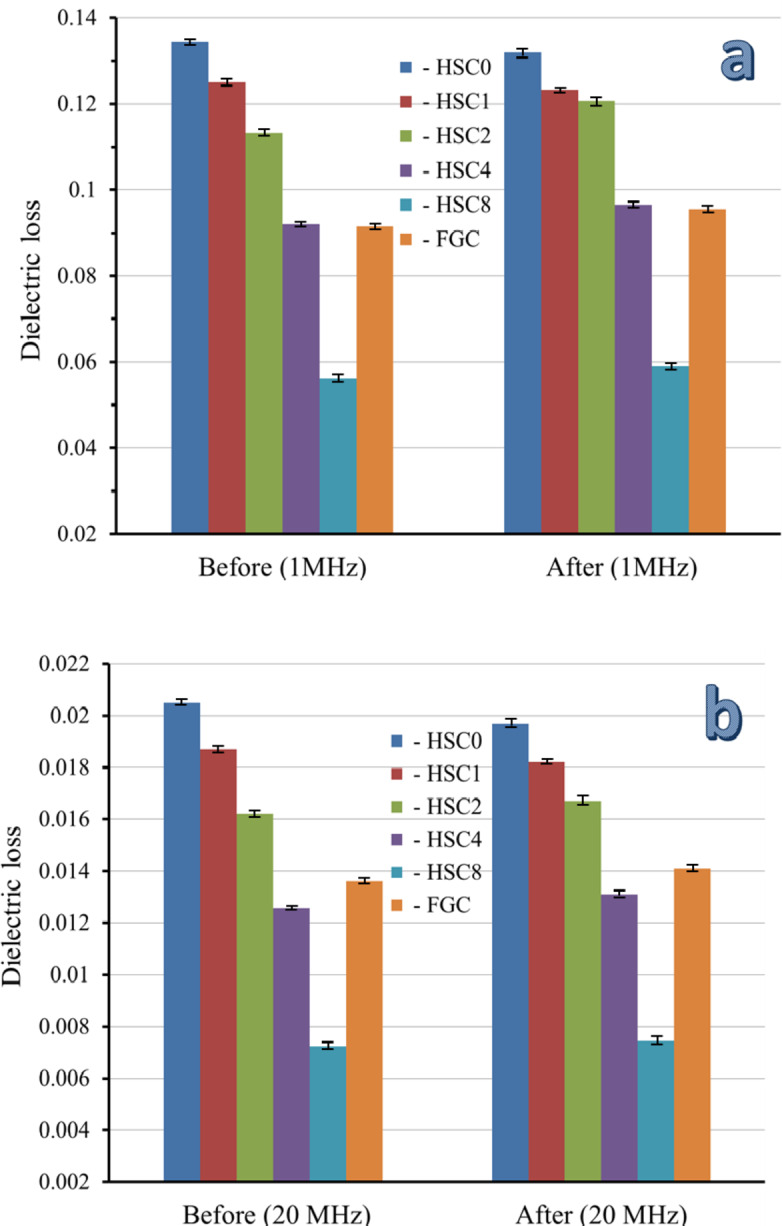
Dielectric loss of the FGC sample and its sintered different layers at (a) 1 MHz and (b) 20 MHz before and after soaking in the SBF solution for fourteen days.

It is impossible to overestimate the significance of biomaterials with favorable electrical properties for promoting bone formation. The electrical and dielectric characteristics of the sintered layers of FGC may be mapped in this work, which is very important for readers interested in biomaterials’ varied properties. As shown in Table 3, measurements of electrical conductivity and dielectric characteristics, including the dielectric constant (ε′) and dielectric loss (ε″), were made at the frequencies of 1, 2, 5, 10, and 20 MHz. Figures 14 and 15 show the AC electrical conductivity and a schematic diagram illustrating the conduction mechanism. On the other hand, Figs. 16 and 17 show the dielectric properties at lower and higher frequencies, i.e., 1 and 20 MHz.

It is evident that when the amounts of CuO and SiC increase and the frequency increases, the samples’ electrical conductivity dramatically improves. Its dielectric qualities are attributed to HA polarization, and the generated polarization significantly facilitates bone tissue healing. The dielectric constant’s actual component, represented by the variable ε′, measures how each energy the material retains as a result of the electric field. Interestingly, this parameter controls the material’s internal dipole alignment. On the other hand, ε″ represents the complex part of the dielectric constant, namely the dielectric loss, which means the energy lost in the dielectric material. The weak oscillation of Ca^2+^, PO_4_^3−,^ and OH^−^ ions is due to the conduction of nanoscale HA at lower frequencies, as in^52^, since the latter ions’ natural frequency coincides with the AC field’s frequency. ε′ changes with a lower frequency due to the oscillations of these dipole moments. Other research has found that HA conductance is only linked to the presence of protons (H)^+^, oxide ions (O^2−^), and lattice OH^−^ ions, which are regarded charge carriers, while Ca^2+^ and PO_4_^3−^ ions do not affect HA conductance^53,54^. The HSC0 sample demonstrates a steady increase with increasing frequency at high frequencies, assuming that the AC conductivity follows the following relationship:

16 $${\sigma _{{\text{ac}}}}\,=\,{\sigma _{{\text{dc}}}}\,+\,{{\text{B}}_{\omega {\text{s}}}}$$

Where *σ*_*dc*_ is the DC electrical conductivity, B is a constant, *ω* is the angular frequency, and s is an exponent^55^.

The segregation of a complex array of ions along the c-axis of the HA crystal structure is responsible for this increase in AC conductivity with increasing frequency^52^. The AC conductivity increases as the amount of SiC and CuO increases because the number of charge carriers increases, lowering the resistance of the FGC^56,57^. The conduction mechanism is summarized in Fig. 15. After evaluating the data, it is evident that ε′ drops as the applied frequency increases while CuO and SiC levels increase. The dipoles in the studied samples tend to orient themselves in the direction of the applied electric field, resulting in a reduction in ε′ values as frequency increases. On the other hand, ε′ records considerably higher values at lower frequencies due to the slow relaxation of the higher-oriented dipoles^52^.

Similarly, increasing the contents of CuO and SiC causes an increase in the content of dipoles that point in the direction of the AC field. With increasing frequency and concentrations of CuO and SiC, a similar declining trend was seen for ε″. The electric dipoles do not have enough time to align themselves with the applied electric field before changing its direction, so there is a notable drop as AC frequency increases. On the other hand, the increasing amounts of CuO and SiC minimize the obtained ε″ values due to the rise in the number of charge carriers^58^. Compared to the literature, adding different contents of CuO and SiC to HA increased the AC conductivity value and decreased the dielectric constant, as seen in Table 4^59–61^.

#### Biological properties of the FGC layers

##### Evaluation of the osseointegration ability

**Fig. 18:**
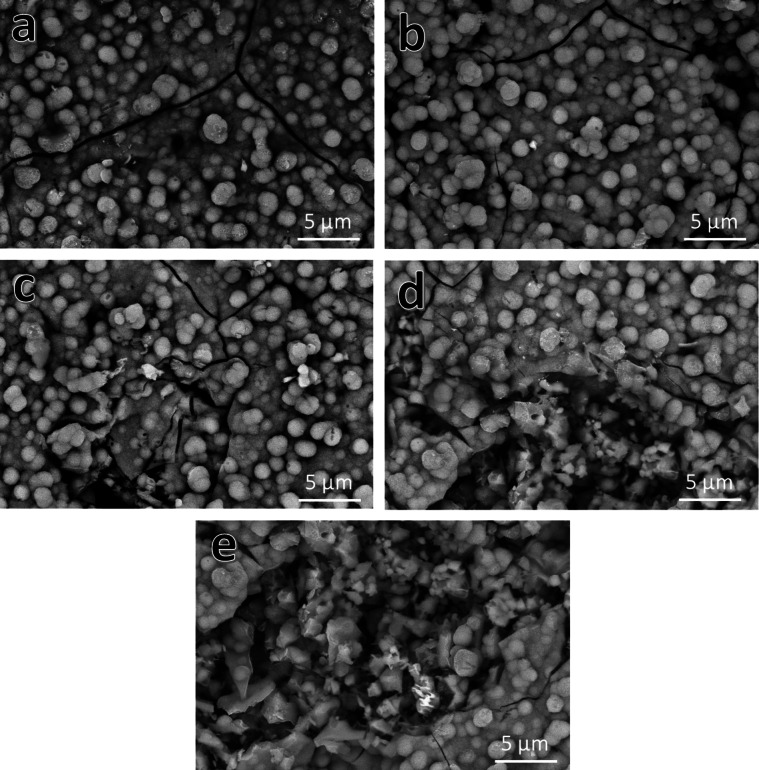
FESEM images of the sintered (a) HSC0, (b) HSC1, (c) HSC2, (d) HSC4, and (e) HSC8 layers of FGC after soaking in SBF solution for fourteen days.

In general, immersion in SBF solution is a simple and reasonably priced test to accurately evaluate a biomaterial’s ability to form a bone-like layer on its surface. Remember that a substance’s “bioactivity property” is its ability to create the desirable layer. Based on this, it is believed that when the material is transplanted into the human body, it will adhere well to the surrounding living bone tissue^62^. All the sintered samples were incubated in SBF solution for fourteen days to test this idea. After that, they were sent to FESEM, as shown in Fig. 18(a-e), to provide the reader with visible evidence of the formation of the HA layer on their surfaces. The sintered samples’ bioactivity exhibits a decreasing sequence, as shown by the following: HSC0 > HSC1 > HSC2 > HSC4 > HSC8. Put another way, the bioactivity of the samples under investigation decreases as the amounts of SiC and CuO increase and the HA concentration decreases. The fact that the layer has formed nearly completely over the HSC0 sample’s surface lends credence to this finding. In contrast, the HSC8 sintered sample does not show full-layer development but has distinct particles visible above the surface. It is anticipated that the loss in forming this desirable surface layer would be negligible since the combined maximum concentration of SiC and CuO does not surpass 12 volume percent. Our latest study^63^ indicates that the quantity of negative charges HA specifically (PO_4_)^3^—determines its bioactivity. These charges can rapidly attach to cations such as (Ca)^2+^ in solution to form an amorphous calcium phosphate layer. Crystallization of the freshly produced layer is then used to construct the HA crystals on the sample surface. In contrast, the implant will be surrounded by fibrous tissue instead of creating a direct bone-implant contact, and the SiC will be coated with a thin coating of silica/silica gel reaction material when exposed to the environment found in the human body. SiC is very strong, resistant to wear and fatigue, chemically stable, and biocompatible, but it cannot create a close bone-bonding contact in vitro. As a result, it has not been employed as a load-bearing bone implant^64^. This prepared FGC^65^ may heal damaged tissues, including those in the knee, hip, teeth, and joints.

##### Correlation between the osseointegration ability of the examined layers and their physical, mechanical, electrical, and dielectric properties

The bulk density and apparent porosity of the FGC sample and its different layers after soaking in SBF solution for 14 days were measured again and represented in Fig. 8(a, b) also to facilitate the discovery of the relationship between the values before and after soaking the samples in the SBF solution for the reader. It can be seen from this figure that the porosity decreased slightly in the first three layers, i.e., HSC0, HSC1, and HSC2 layers, after immersion in the SBF solution. On the other hand, this decrease is barely seen in the HSC4 and HSC8 layers. Naturally, the FGC sample shows a trend similar to all its examined layers.

The reason for obtaining these results is that the HSC1, HSC2, and HSC4 layers formed a good apatite layer on their surfaces, which contributed to closing the pores and reducing the porosity values. However, the apatite layer formed on the surface of the HSC4 and HSC8 layers is less dense than that which appeared on the surface of the other layers, which led to a slight decrease in the porosity values in these samples.

To study the effect of the bone-like layer formed on the surfaces of the sintered FGC sample and its layers on their mechanical properties, i.e., microhardness, ultimate strength, and set of elastic moduli of all samples were measured after soaking in SBF solution for 14 days and are represented in Figs. 9 and 11, and 12. As shown from these figures, one can observe that all mechanical properties of the FGC and its layers are almost not affected after their soaking in the SBF solution. It is important to note that these results are encouraging because they mean that the implanted area is stable, which reduces the risk of fractures or damage to the implants on one side. On the other side, enhancing the durability of implants by maintaining their properties after insertion reduces the possibility of corrosion or chemical interactions with body fluids, thus prolonging the implant’s life and reducing the need for replacement or further surgical procedures.

The electrical and dielectric properties of the FGC sample and all its sintered layers were examined after incubation in SBF solution at the same frequencies that were used before soaking the samples in the SBF to study the effect of the surface apatite layer produced, and the results are summarized in Table 5. Also, these properties measured at 1 and 20 MHz are represented in Figs. 14 and 16, and 17 to facilitate comparison between the results obtained before and after soaking in the SBF solution.

After soaking in SBF solution, the HSC0 and HSC1 samples had higher AC conductivity. This result is closely related to the results discussed in the previous section, as these samples show good bioactivity and a dense surface layer apatite that closes the pores and thus increases the electrical conductivity, bearing in mind that this increase in the AC conductivity of HSC1 layer is less pronounced compared to the HSC0 layer due to that the apatite surface layer is less than that appear in the HSC0. On the contrary, the electrical conductivity of the remaining layers, i.e., HSC2, HSC3, and HSC4, gradually decreases after soaking in the SBF solution. This decrease can be explained by forming a surface apatite layer that impedes the flow of electrical charges. Keep in mind that as this surface layer decreases, the change in AC conductivity after soaking in the SBF solution also decreases. As expected, these changes in AC conductivity of the samples before and after soaking in SBF solution become more pronounced at the highest frequency, i.e., 20 MHz. Finally, ε′ and ε″ follow an opposite trend seen by the AC conductivity, where they decrease after soaking the HSC0 and HSC1 layers in SBF solution and increase in the case of the remaining layers. As mentioned above, the findings indicated that the electrical and dielectric properties of biomaterials post-implantation into human tissues and following exposure to physiological fluids differ from those measured before implantation, thereby necessitating an investigation of these properties before and after the intervention.

One of this manuscript’s most important focuses is monitoring the prepared biomaterials’ electrical and dielectric properties. We have previously mentioned in this manuscript the importance of electrical properties in improving bone healing. Here in this section, we will mention the biological significance of materials with appropriate dielectric properties in orthopedic treatment, which can be summarized as follows^66,67^:

- Protein adsorption, which promotes cell attachment and proliferation, is being optimized.
- A key component of osseointegration is the influence of cellular activity, including osteoblast adhesion, differentiation, and proliferation.
- By stimulating the piezoelectric characteristics of bone, conductive and dielectric materials may improve communication between the implant and the surrounding bone tissue.
- They affect the development of a biological apatite layer by promoting or inhibiting ionic transport at the interface.
- For bone growth and integration, interaction with osteogenic cells is essential.

##### Evaluation of the antibacterial properties of the sintered layers

**Fig. 19:**
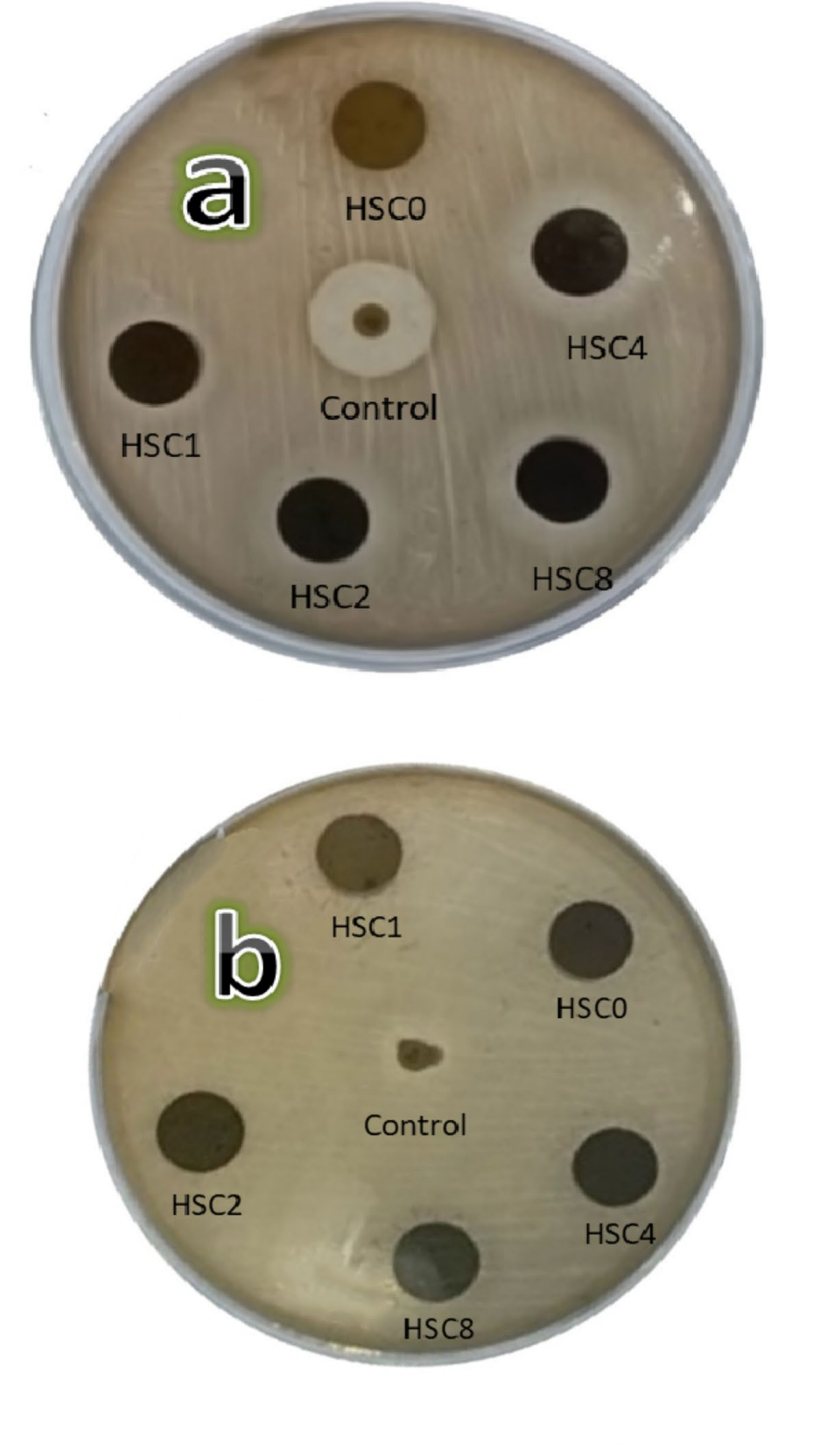
Photos of Petri dishes after conducting agar disc-diffusion assays against (a) *S. aureus* and (b) *E. coli* for sintered FGC layers.

Recently, there has been a lot of interest in incorporating antimicrobial metals (such as silver, copper, and zinc) into biomaterials to decrease and/or prevent bacterial infection caused by surgical procedures^68^. In this study, the antibacterial activity of the prepared materials against *S. aureus* (ATCC 6538) and *E. coli* (ATCC 25922) as Gram + and Gram − bacteria was investigated using agar disc diffusion assays, as shown in Fig. 19(a, b). The key to selecting these species is that they are one of the most common causes of nosocomial infections that can be treated with broad-spectrum antibiotics. Besides, they can cause severe complications following a surgical procedure. Table 6 shows the diameters of inhibition zones around the tested materials as determined by the agar diffusion test. Except for the HSC0 sample, all samples showed a good antibacterial effect against *S. aureus*, considering that successive CuO content increased this effect.

On the other hand, only HSC4 and HSC8 samples showed significant antibacterial effects against *E. coli*. It is essential to highlight that even the antibiotic used in this experiment showed low inhibitory strength against *E. coli* bacteria. Notably, the antibacterial impact of Cu can be influenced by various factors. The liberated Cu^2+^ ions interact with –SH_2_ and –NH_2_ of proteins on bacteria’s cell membranes, causing severe disruption in cellular metabolism and the death of the bacteria.

Moreover, Cu^2+^ can bind with DNA molecules, breaking their spiral shape and eventually resulting in cell death. Furthermore, since it can transition between Cu^+^ and Cu^2+^, Cu is widely recognized for its ability to act as an electron donor/acceptor. This desirable characteristic is very detrimental to the bacterial cell because it generates reactive OH^–^ radicals that oxidize proteins and lipids, resulting in serious problems for the bacterial cell^69^. It is also important to note that excessive Ca^2+^ ion release significantly disrupts the potential of the bacterial cell membrane, leading to drastic changes in osmotic pressure and, ultimately, the death of the bacterial cell^70^. These findings align with previous research^71,72^.

##### Evaluation of the biocompatibility of the sintered layers

The capacity of a biomaterial to carry out its intended function in a particular application without triggering allergic or inflammatory responses is known as biocompatibility. “Host” refers to the live creature or system interacting with the biomaterial; “host response” refers to the host’s reaction to a foreign material. Table 7 summarizes the proportion of dead cells brought on by the HSC0, HSC2, and HSC8 layers and the survivability of osteosarcoma cells in contact with these layers. 1.2%, 4.7%, and 8.3% of cells died in contact with three layers, respectively. These results imply that the materials under Evaluation are safe for use in bone replacement applications and show high biocompatibility.

## Conclusions

Five layers of functionally graded composite (FGC) were created for this investigation. Copper oxide (CuO), silicon carbide (SiC), and hydroxyapatite (HA) were present in varying amounts in each layer. The leading cause of the improvements in microhardness, ultimate strength, Young’s modulus, longitudinal modulus, bulk modulus, and shear modulus by 36%, 29.48%, 35.29%, 44.44%, 41.17%, and 11.47%, respectively, in comparison to the sample containing only -, was the increase in SiC and CuO contents to 4 and 8 vol%, respectively. According to field emission scanning electron microscopy (FESEM), the samples immersed in simulated body fluid (SBF) developed a layer resembling bone on their surfaces. This layer showed that the samples may osseointegrate if implanted within the human body. CuO at eight vol% into the FGC layer inhibited *S. aureus* and *E. coli* bacterial growth up to 23 and 16 mm, respectively. Thankfully, the proportion of dead bone cells in contact with this sample was only 8.3%, reflecting its high biocompatibility. The electrical characteristics of the sintered layers were improved up to 4.39 × 10^− 4^ S/cm by a mixture of SiC and CuO, which aids in the repair of bone fractures. Additionally, immersion in the SBF solution did not substantially change all mentioned material properties of the FGC layers. This positive result implies that these characteristics won’t change if the samples are implanted into a human body, proving their stability. The wear rate of the samples improved by increasing CuO and SiC, reaching 5 × 10^− 5^, 1 × 10^− 4^, and 1.5 × 10^− 4^ mg/s, even with applying loads of 10, 20, and 40 N, respectively. The findings show that the FGC and its layers are excellent. However, it is hard to suggest for this application because this suggestion relies on the surgeon’s view. Some think samples with intense antibacterial or biological activity are essential. Some argue that samples with incredible electrical characteristics are more critical because they encourage bone cells to form new bones.

**Table 1:** Scheme of the chemical composition of prepared layers for FGC (vol%).

Sample code	Hydroxyapatite (HA)	Silicon carbide (SiC)	Copper oxide (CuO)
HSC0	100	0	0
HSC1	98.5	0.5	1
HSC2	97	1	2
HSC4	94	2	4
HSC8	88	4	8

**Table 2:** Human blood plasma and SBF solution ion concentrations (mM).

Solution	Ion concentration (mM)							
	Na^+^	K^+^	Mg^2+^	Ca^2+^	Cl^−^	HCO_3_^−^	HPO_4_^2−^	SO_4_^2−^
SBF	142.0	5.0	1.5	2.5	147.8	4.2	1.0	0.5
Blood plasma	142.0	5.0	1.5	2.5	103.0	27.0	1.0	0.5

**Table 3:** All tested samples’ AC conductivity and dielectric properties were measured for the FGC sample and its layers at various frequencies, i.e., 1, 2, 5, 10, and 20 mhz.

Samples code		1 MHz	2 MHz	5 MHz	10 MHz	20 MHz
		Electrical conductivity (S/cm) ± 0.002 × 10^− 6^				
HSC0		1.52 × 10^− 6^	2.22 × 10^− 6^	3.53 × 10^− 6^	5.29 × 10^− 5^	1.60 × 10^− 4^
HSC1		1.82 × 10^− 6^	2.48 × 10^− 6^	3.66 × 10^− 6^	5.61 × 10^− 5^	2.02 × 10^− 4^
HSC2		2.26 × 10^− 6^	2.74 × 10^− 6^	3.88 × 10^− 6^	6.02 × 10^− 5^	2.45 × 10^− 4^
HSC4		3.49 × 10^− 6^	5.62 × 10^− 6^	4.48 × 10^− 6^	6.78 × 10^− 5^	3.15 × 10^− 4^
HSC8		4.82 × 10^− 6^	6.31 × 10^− 6^	6.09 × 10^− 6^	8.12 × 10^− 5^	4.39 × 10^− 4^
FGC		3.17 × 10^− 6^	3.54 × 10^− 6^	4.55 × 10^− 6^	5.21 × 10^− 5^	3.12 × 10^− 4^
		Dielectric constant ± 0.002				
HSC0		6.556	6.202	5.113	3.721	1.922
HSC1		6.326	5.940	4.965	3.575	1.851
HSC2		5.934	5.610	4.657	3.398	1.779
HSC4		5.179	4.978	3.744	2.697	1.651
HSC8		3.939	3.993	2.265	1.594	1.454
FGC		5.179	4.890	3.030	2.384	1.511
		Dielectric loss ± 0.002				
HSC0		0.134	0.114	0.099	0.066	0.021
HSC1		0.125	0.106	0.087	0.062	0.019
HSC2		0.113	0.098	0.079	0.060	0.016
HSC4		0.092	0.077	0.062	0.051	0.013
HSC8		0.056	0.049	0.039	0.032	0.007
FGC		0.092	0.086	0.072	0.051	0.014

**Table 4:** The composition, AC electrical conductivity, and dielectric properties of various compositions^58–60^.

Composition	AC conductivity	Dielectric constant	Dielectric loss
HA/Barium titanate	2.5 × 10^–10^ (Ω^-1^.cm ^-1^)	15.5	….
HA/Na_0.5_K_0.5_NbO_3_	2.686 × 10^− 8^ (Ω^-1^.cm ^-1^)	29.81	….
HA/Cordierite/zirconia	1.08 × 10^− 7^ (s/cm)	14.54	0.397

**Table 5:** All samples’ AC conductivity and dielectric properties of the FGC sample and its layers after 14 days of incubation in SBF solution and examination at various frequencies, i.e., 1, 2, 5, 10, and 20 mhz.

Samples code		1 MHz	2 MHz	5 MHz	10 MHz	20 MHz
		Electrical conductivity (S/cm) ± 0.002 × 10^− 6^				
HSC0		1.65 × 10^− 6^	2.30 × 10^− 6^	3.66 × 10^− 6^	5.45 × 10^− 5^	1.71 × 10^− 4^
HSC1		1.89 × 10^− 6^	2.54 × 10^− 6^	3.73 × 10^− 6^	5.67 × 10^− 5^	2.07 × 10^− 4^
HSC2		2.03 × 10^− 6^	2.59 × 10^− 6^	3.74 × 10^− 6^	6.78 × 10^− 5^	2.32 × 10^− 4^
HSC4		3.28 × 10^− 6^	3.49 × 10^− 6^	4.38 × 10^− 6^	6.63 × 10^− 5^	3.06 × 10^− 4^
HSC8		4.68 10^− 6^	2.55 × 10^− 6^	6.03 × 10^− 6^	8.02 × 10^− 5^	4.34 × 10^− 4^
FGC		2.99 × 10^− 6^	3.41 × 10^− 6^	4.34 × 10^− 6^	5.04 × 10^− 5^	3.07 × 10^− 4^
		Dielectric constant ± 0.002				
HSC0		6.406	5.950	4.942	3.641	1.892
HSC1		6.241	5.791	4.885	3.525	1.822
HSC2		6.134	5.786	4.779	3.511	1.853
HSC4		5.336	5.113	3.861	2.764	1.700
HSC8		4.054	4.113	2.325	1.634	1.487
FGC		5.289	5.018	3.140	2.442	1.571
		Dielectric loss ± 0.002				
HSC0		0.132	0.110	0.097	0.064	0.020
HSC1		0.123	0.103	0.086	0.060	0.018
HSC2		0.121	0.100	0.082	0.063	0.017
HSC4		0.095	0.080	0.064	0.050	0.013
HSC8		0.059	0.055	0.040	0.032	0.007
FGC		0.096	0.091	0.074	0.052	0.014

**Table 6:** The measured Inhibition zone diameters for all examined layers of FGC against *S. aureus* and *E. coli* bacteria.

Layers’ code	Inhibition zone (mm)	
	*S. aureus*	*E. coli*
HSC0	Nil	Nil
HSC1	17	Nil
HSC2	19	Nil
HSC4	21	15
HSC8	23	16

Nil means no antibacterial effect due to the absence of an inhibition zone surrounding the tested sample.

**Table 7:** The samples that were analyzed with human osteosarcoma cell lines.

Sample code	IC_50_ (µg/ml)	IC_90_ (µg/ml)	Remarks
HSC0	………………	………………….	1.2% at 100 ppm
HSC2	………………	………………….	4.7% at 100 ppm
HSC8	………………	………………….	8.3% at 100 ppm

IC50: The sample’s lethal dose at which 50% of cells die within 48 h.

IC90: The sample’s lethal concentration results in 90% of cells dying in 48 h.

## The source of cells used in this manuscript

The human osteosarcoma Saos-2 cell line used in this study was obtained from ATCC, USA, under the Bioassay-Cell Culture Laboratory of the National Research Centre in Egypt.

## Data Availability

The datasets generated and/or analyzed during the current study are not publicly available because all data are presented in the article and therefore, there is no need to include raw data but they are available from the corresponding author upon reasonable request.
